# PTEN is a predictive biomarker of trastuzumab resistance and prognostic factor in HER2-overexpressing gastroesophageal adenocarcinoma

**DOI:** 10.1038/s41598-021-88331-3

**Published:** 2021-04-27

**Authors:** Daiju Yokoyama, Shigeo Hisamori, Yasunori Deguchi, Tatsuto Nishigori, Hiroshi Okabe, Seiichiro Kanaya, Dai Manaka, Yoshio Kadokawa, Hiroaki Hata, Sachiko Minamiguchi, Shigeru Tsunoda, Kazutaka Obama, Yoshiharu Sakai

**Affiliations:** 1 Department of Surgery, Graduate School of Medicine, Kyoto University, 54, Shogoin-Kawahara-cho, Sakyo-ku, Kyoto, 606-8507 Japan; 2 Department of Surgery, Ijinkai Takeda General Hospital, 28-1, Ishida Moriminami-cho, Fushimi-ku, Kyoto, 601-1495 Japan; 3 Department of Gastroenterological Surgery, New Tokyo Hospital, 1271, Wanagaya, Matsudo, Chiba 270-2232 Japan; 4 Department of Surgery, Osaka Red Cross Hospital, 5-30, Fudegasaki-cho, Tennoji-ku, Osaka, 543-8555 Japan; 5 Department of Surgery, Kyoto Katsura Hospital, 17, Yamada-Hirao-cho, Nishikyo-ku, Kyoto, 615-8256 Japan; 6 Department of Gastrointestinal Surgery, Tenri Hospital, 200, Mishima-cho, Tenri, Nara 632-8552 Japan; 7 Department of Surgery, National Hospital Organization Kyoto Medical Center, 1-1, Fukakusa-Mukaihata-cho, Fushimi-ku, Kyoto, 612-8555 Japan; 8 Department of Diagnostic Pathology, Graduate School of Medicine, Kyoto University, 54, Shogoin-Kawahara-cho, Sakyo-ku, Kyoto, 606-8507 Japan

**Keywords:** Cancer, Biomarkers, Gastroenterology, Oncology

## Abstract

Poor trastuzumab (Tmab) response of patients with human epidermal growth factor receptor 2-overexpressing gastric or gastroesophageal junction adenocarcinoma (HER2-GEA) is associated with the inhibition of phosphatase and tensin homolog (PTEN) expression. In this multicenter, retrospective observational study, pathological samples of patients with HER2-GEA receiving Tmab-combined chemotherapy were immunohistochemically analyzed for PTEN expression. The primary endpoints were disease control rate (DCR), progression-free survival (PFS), and overall survival (OS). We assessed the effect of conventional chemotherapy and Tmab alone or combined with PI3K pathway inhibitors in vitro in HER2-GEA cells with or without PTEN expression. Twenty-nine and 116 patients were in the PTEN-loss and PTEN-positive groups, respectively. In patients with the target region, DCR was significantly lower in PTEN-loss patients than in PTEN-positive patients (67% and 87%, respectively, p = 0.049). The multivariate analysis demonstrated that PTEN loss was significantly associated with shorter PFS (HR = 1.63, p = 0.035) and OS (HR = 1.83, p = 0.022). PTEN knockdown did not affect the cytostatic effect of 5-FU and cisplatin, whereas Tmab combined with the PI3K/mTOR inhibitor NPV-BEZ235 suppressed PTEN-knockdown cell proliferation. In patients with HER2-GEA, PTEN loss is a predictive biomarker of Tmab resistance and prognostic factor. Molecular-targeted therapy with a PI3K/mTOR inhibitor would be effective for HER2-GEA with PTEN loss.

## Introduction

Gastric cancer, including gastroesophageal junction adenocarcinoma (GEA), is the fifth most frequently diagnosed malignancy and the third leading cause of cancer-related death worldwide^1^. Several patients with GEA have metastatic disease at diagnosis, and the tumor is considered unresectable oncologically; systemic chemotherapy for GEA with distant metastasis or recurrence results in a poor prognosis^2^.

The identification of human epidermal growth factor receptor 2 (HER2) as an oncogene has enabled the development of molecular-targeted therapies for GEA and breast cancer^3–7^. HER2 amplification and overexpression lead to continuous stimulation of the downstream phosphatidylinositol-3 kinase (PI3K) or mitogen-activated protein kinase (MAPK) pathway, triggering uncontrolled cell proliferation, invasiveness, apoptosis resistance, and angiogenesis^8^. Systemic chemotherapy combined with trastuzumab (Tmab) demonstrated a significant survival benefit in an international, randomized controlled trial and was recommended for unresectable or recurrent HER2-overexpressing GEA (HER2-GEA)^9^. However, the response rate was only 47%, indicating that 53% of the patients did not have an objective clinical response to Tmab despite HER2 overexpression. Moreover, most patients had intrinsic or developed acquired Tmab resistance within 1 year. Thus, elucidating the molecular mechanisms and identifying a predictive biomarker for Tmab resistance are critical for improving the survival of patients with HER2-GEA.

In breast cancer, constitutive activation of the PI3K pathway due to phosphatase and tensin homolog (PTEN) deficiency is a major Tmab resistance mechanism^10–12^. By applying this concept to gastric cancer, we recently reported the association between low PTEN expression and Tmab resistance in HER2-GEA^13^. In this previous study, approximately one-third of patients with advanced HER2-GEA who underwent gastrectomy showed PTEN loss, which tended to decrease the clinical objective response to Tmab. Moreover, we showed that Tmab-induced growth suppression, apoptosis, and G1 cell cycle arrest were inhibited by PTEN knockdown using gastric or gastroesophageal adenocarcinoma cell lines. These results indicated that PTEN loss could be a predictive indicator for poor Tmab response, but this association has not been assessed in a large cohort of patients with HER2-GEA.

Several molecule types have been suggested as potential therapeutics for the Tmab resistant subgroup in breast cancer^14,15^. These molecules potentially inhibit PI3K pathway activation by targeting one or more of its enzymes, including PI3K, protein kinase B (Akt), mammalian target of rapamycin complex 1 (mTORC1), and mTORC2, which intervene upstream or downstream of PTEN^16^. In the BOLERO-3 trial, an international, randomized controlled trial, Tmab was used in combination with the mTORC1 inhibitor everolimus, which significantly prolonged progression-free survival (PFS) of patients with Tmab-resistant HER2-positive advanced breast cancer^17^. Furthermore, the GRANITE-1 study investigated the efficacy and safety of everolimus in patients with advanced gastric cancer that progressed after one or two lines of prior systemic chemotherapy^18^. In this study, everolimus did not improve the overall survival (OS) of patients with advanced gastric cancer, compared with that achieved by best supportive care, but a subgroup analysis has not been reported. It is still unclear whether mTOR inhibitors are effective in patients with HER2-overexpressing and Tmab-resistant gastric cancer. To date, there are no reports on other molecular therapies inhibiting the PI3K pathway in this patient subgroup. In this retrospective multicenter study, we investigated the potential of PTEN loss as a predictive biomarker of Tmab response in patients with HER2-GEA and explored a new molecular treatment option targeting the signal component of the PI3K pathway.

## Results

### PTEN loss is associated with a poor clinical response to Tmab-CTx in patients with HER2-GEA

A total of 174 patients with HER2-GEA were treated with Tmab-combined chemotherapy (Tmab-CTx) from January 2011 to December 2016 in six hospitals, including the Kyoto University Hospital. We excluded 23 patients whose detailed clinical information or pathological tissue samples were not available and 5 patients who were not subjected to a clinical objective evaluation based on Response Evaluation Criteria in Solid Tumors (RECIST version 1.1)^19^. One patient who was not evaluated to have HER2 2+ or 3+ in the subsequent assessment was also excluded. Finally, 145 patients were enrolled in this study (Table 1). The PTEN-loss group had 29 patients and the PTEN-positive group had 116 patients.

**Table 1:**
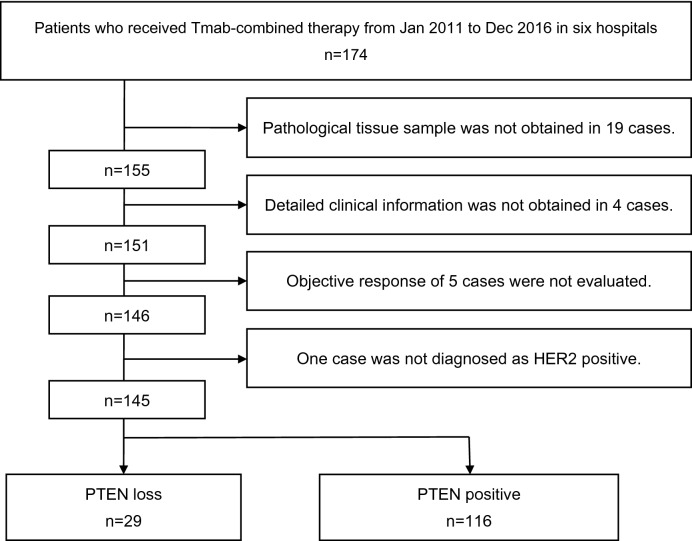
Flow diagram of study selection. One hundred forty-five patients were assigned to either the PTEN-loss or PTEN-positive group, based on the PTEN IHC results. The PTEN-loss group had 29 patients and the PTEN-positive group had 116 patients.

An assessment of the patient characteristics showed no significant differences in age, sex, Charlson comorbidity index (CCI), ECOG-PS, primary site, Lauren classification, macroscopic type, HER2 score, or metastatic situation between the two PTEN expression status groups, whereas the BMI was significantly lower in the PTEN-loss group (p = 0.041) than in the PTEN-positive group (Table 2A). The proportion of patients who received previous chemotherapy did not significantly vary, but the PTEN-positive group had a significantly lower proportion of patients who underwent gastrectomy than the PTEN-loss group (66% vs. 22%, p < 0.001).

**Table 2:** Characteristics of patients and chemotherapy regimen with trastuzumab.

(A)				
Variables		PTEN loss	PTEN positive	p value^a^
		n = 29	n = 116	
		n	n	
Age, years	Mean (SD)	68.4 (10.8)	68.2 (8.9)	0.915
Sex	Male	21 (72%)	81 (70%)	1.000
	Female	8 (28%)	35 (30%)	
BMI	Mean (SD)	19.5 (3.2)	21 (3.5)	0.041
Charlson Comorbidity Index	0 or 1	23 (79%)	99 (85%)	0.407
	2 ≤	6 (21%)	17 (15%)	
ECOG-PS	0 or 1	22 (76%)	86 (74%)	1.000
	2 or 3	7 (24%)	30 (26%)	
Primary site	Stomach	27 (93%)	113 (97%)	0.261
	GE junction	2 (7%)	3 (3%)	
Lauren classification	Intestinal	15 (52%)	53 (46%)	0.692
	Diffuse	8 (28%)	30 (26%)	
	Mixed	6 (21%)	33 (28%)	
Macroscopic type	Type 4	1 (3%)	14 (12%)	0.305
	Other	28 (97%)	102 (88%)	
HER2 score	2+	4 (14%)	20 (17%)	0.785
	3+	25 (86%)	96 (83%)	
Liver metastasis	Yes	9 (31%)	49 (42%)	0.298
Lung metastasis	Yes	5 (17%)	21 (18%)	1.000
Para-aorta LN metastasis	Yes	10 (34%)	45 (39%)	0.831
Bone metastasis	Yes	0 (0%)	9 (8%)	0.203
peritoneal dissemination	Yes	14 (48%)	34 (29%)	0.076
Number of metastatic sites	0–1	18 (62%)	73 (63%)	1.000
	2 ≤	11 (38%)	43 (37%)	
Previous chemotherapy	Yes	8 (28%)	16 (14%)	0.094
Previous gastrectomy	Yes	19 (66%)	26 (22%)	< 0.001

(B)				
Variable	PTEN loss	PTEN positive	p value^a^	
	n = 29	n = 116		
	n	n		
**Regimen**				
Trastuzumab alone	2 (7%)	1 (1%)	0.102	
5-FU or S-1 or Cape	22 (76%)	108 (93%)	0.013	
CDDP or L-OHP	19 (66%)	103 (89%)	0.004	
Others^b^	6 (21%)	10 (9%)	0.092	

(A) Characteristics of patients divided according to the phosphatase and tensin homolog (PTEN) status. (B) Chemotherapy regimen with trastuzumab.

*PTEN* phosphatase and tensin homolog, *GE* gastroesophageal, *HER2* human epidermal growth Factor Type2, *5-FU* fluorouracil, *S-1* tegafur/gimeracil/oteracil, *Cape* capecitabine, *CDDP* cisplatin, *L-OHP* oxaliplatin.

^a^Fisher’s exact test and Student’s *t* test were used for categorical items and continuous variables, respectively.

^b^Others include docetaxel, paclitaxel, and irinotecan.

Tmab is primarily administered in combination with other anticancer agents, which were classified in our Tmab-CTx study as fluoropyrimidine antimetabolites, platinum-based drugs, and others (Table 2B). Fluoropyrimidine antimetabolites included 5-fluorouracil (5-FU), tegafur/gimeracil/oteracil (S-1), and capecitabine (Cape). Platinum-based drugs included cisplatin (CDDP) and oxaliplatin (L-OHP). The other drugs included docetaxel (DOC), paclitaxel (Pac), and irinotecan (CPT-11). The proportion of patients on Tmab-CTx using fluoropyrimidine antimetabolites and platinum-based drugs was significantly higher in the PTEN-positive group than in the PTEN-loss group (p = 0.013 and 0.004, respectively).

An assessment of the objective clinical response to Tmab-CTx for GEA indicated that the disease control rate (DCR) was lower in the PTEN-loss group than in the PTEN-positive group (72.4% and 86.2%, respectively; p = 0.094), whereas there was no significant difference in the response rate between the PTEN-loss and PTEN-positive groups (34.5% and 44.8%, respectively; p = 0.402) (Table 3A). Among the patients with target lesions, DCR was significantly lower in the PTEN-loss group than in the PTEN-positive group (66.7% and 86.6%, respectively; p = 0.049), whereas the response rate did not differ between the PTEN-loss and PTEN-positive groups (47.6% and 52.6%, respectively; p = 0.811) (Table 3B). The PTEN-loss group had a relatively shorter stable disease (SD) duration than the PTEN-positive group (8.3 months vs. 13.6 months, respectively; p = 0.063) (Table 3C).

**Table 3:** Clinical response to trastuzumab combined chemotherapy.

(A)			
Variables	PTEN loss	PTEN Positive	p value^a^
	n = 29	n = 116	
	n	N	
**Clinical response**			0.114
CR	0 (0%)	3 (3%)	
PR	10 (34%)	49 (42%)	
SD	4 (14%)	33 (28%)	
Non-CR/non-PD	7 (24%)	15 (13%)	
PD	8 (28%)	16 (14%)	
Disease control rate^b^	21 (72%)	100 (86%)	0.094
Response rate^c^	10 (34%)	52 (45%)	0.402

(B)			
Variables	PTEN loss	PTEN Positive	p value^a^
	n = 21	n = 97	
	n	N	
**Clinical response**			0.142
CR	0 (0%)	2 (2%)	
PR	10 (48%)	49 (51%)	
SD	4 (19%)	33 (34%)	
PD	7 (33%)	13 (13%)	
Disease control rate^d^	14 (67%)	84 (87%)	0.049
Response rate^c^	10 (48%)	51 (53%)	0.811

(C)			
Variables	PTEN loss	PTEN positive	p value^a^
	Months	Months	
**Duration of stable disease**^**e**^			
Mean (SD)	8.3 (7.9)	13.6 (14.7)	0.063

(A). Objective response rate and disease control rate in 145 patients. (B). Objective response rate and disease control rate in 118 patients with target lesions. (C). Duration of stable disease.

*CR* complete response, *PR* partial response, *SD* stable disease, *PD* progressive disease.

^a^Fisher extract test was used for categorical items and Student’s *t* test was used for continuous variables, respectively.

^b^Disease control rate: the sum of the proportion of CR and PR and SD and non-CR/non-PD in (A).

^c^Response rate: the proportion of complete response and partial response in (A) and (B).

^d^Disease control rate: the sum of the proportion of CR and PR and SD in (B).

^e^Duration of stable disease: the duration from the date when trastuzumab-combined therapy was first administered to the date when PD was determined.

### PTEN loss is associated with a significantly shorter PFS and OS in patients with HER2-GEA receiving Tmab-CTx

**Figure 1:**
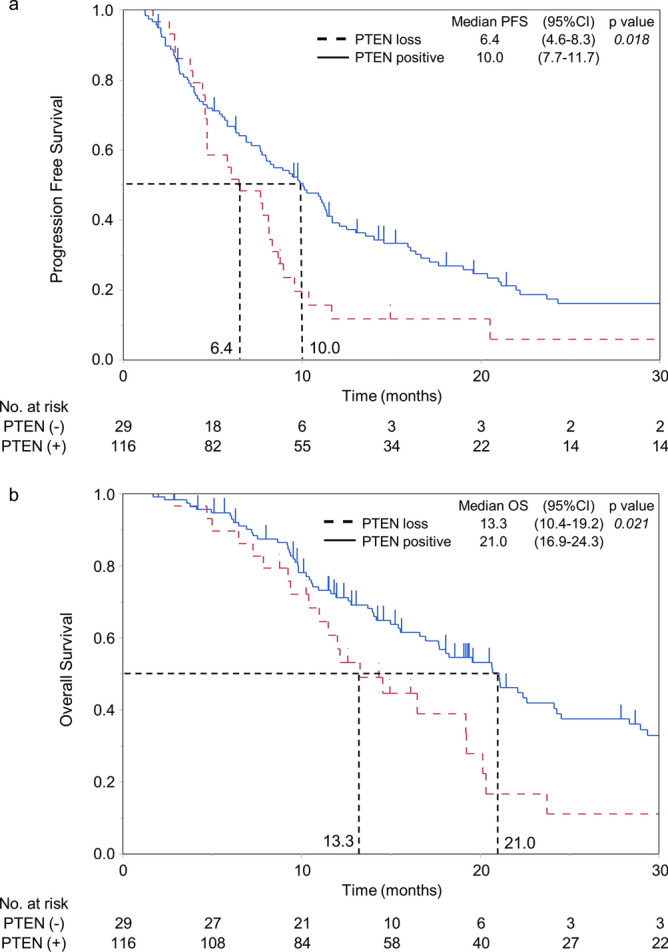
Overall survival (OS) and progression-free survival (PFS) of Tmab-combined chemotherapy (Tmab-CTx). (**a**) OS was calculated from the date when Tmab-based therapy was first administered. (**b**) PFS was defined as the period from the date when Tmab-CTx was first administered to the date when an objective evaluation as “progression” was determined from the review of the patient chart or to patient’s death. Survival curves were obtained using the Kaplan–Meier method and analyzed using the log-rank test.

With an overall median follow-up duration of 14.3 months, the median PFS and OS of all patients with HER2-GEA were 8.9 and 19.2 months, respectively. Patients with PTEN loss had a significantly shorter PFS period than those who were PTEN positive (Fig. 1a; 6.4 vs. 10.0 months, respectively; p = 0.018). Furthermore, PTEN-loss patients had a significantly shorter OS than PTEN-positive patients (Fig. 1b; 13.3 vs. 21.0 months, respectively; p = 0.021).

### PTEN loss has prognostic significance and is a predictive factor for shorter OS and PFS in patients with HER2-GEA receiving Tmab-CTx

To identify predictive factors for OS and PFS in patients receiving Tmab-CTx, we subjected various clinicopathological and molecular characteristics to univariate and multivariate analyses (Table 4). We found that two or more sites of metastases and PTEN loss were significantly related to shorter PFS in patients with HER2-GEA based on the univariate analysis (p = 0.003 and p = 0.020, respectively) and multivariate analysis (p = 0.002 and p = 0.035, respectively). For OS, non-platinum containing regimen, two or more sites of metastases, and PTEN loss were significantly related to shorter OS in patients with HER2-GEA based on the univariate analysis (p = 0.049, p = 0.002, and p = 0.023, respectively). In the multivariate analysis, macroscopic type 4, two or more sites of metastases, and PTEN loss were significantly related to shorter OS (p = 0.038, 0.001, and 0.022, respectively).

**Table 4:** Univariate and multivariate analyses of overall survival progression free survival.

(A)					
	Progression free survival				
	Univariate analysis			Multivariate analysis	
	Median survival^a^	HR (95% CI)	p value^b^	HR (95% CI)	p value^b^
Age (> 65 vs. ≤ 65) years	8.4 vs. 9.6	1.05 (0.70–1.56)	0.827		
Sex (Male vs. Female)	9.3 vs. 8.0	0.97 (0.65–1.44)	0.870		
BMI (≤ 18.5 vs. > 18.5)	8.4 vs. 8.9	1.16 (0.77–1.72)	0.479		
Charlson Comorbidity Index (2 ≤ vs. 0 or 1)	10.4 vs. 8.4	0.99 (0.61–1.63)	0.984		
Lauren classification (diffuse or Mixed vs. intestinal)	8.4 vs. 8.9	0.98 (0.68–1.42)	0.922		
Macroscopic type (type 4 vs. other)	5.0 vs. 8.9	1.39 (0.78–2.48)	0.263		
HER2 score (3 + vs. 2 +)	8.7 vs. 8.4	0.85 (0.52–1.38)	0.503		
Regimen (Platinum drug ( +) vs. Platinum drug (–))	9.5 vs. 5.8	0.68 (0.42–1.10)	0.119	0.72 (0.36–1.46)	0.366
Number of metastatic sites (2 ≤ vs. 0–1)	7.6 vs. 10.3	1.77 (1.22–2.56)	0.003	1.80 (1.23–2.62)	0.002
Previous chemotherapy (yes vs. no)	6.0 vs. 9.3	1.40 (0.87–2.24)	0.165	0.99 (0.50–1.97)	0.983
Previous gastrectomy (yes vs. no)	6.5 vs. 9.8	1.21 (0.82–1.78)	0.339	–	
PTEN (loss vs. positive)	6.4 vs. 10.0	1.70 (1.09–2.65)	0.020	1.63 (1.04–2.57)	0.035

(B)					
	Overall survival				
	Univariate analysis			Multivariate analysis	
	Median survival^a^	HR (95% CI)	p value^b^	HR (95% CI)	p value^b^
Age (> 65 vs. ≤ 65) years	20.6 vs. 17.7	0.96 (0.61–1.51)	0.863		
Sex (Male vs. Female)	19.6 vs. 16.9	0.98 (0.62–1.56)	0.937		
BMI (≤ 18.5 vs. > 18.5)	17.7 vs. 20.3	1.24 (0.79–1.94)	0.349		
Charlson Comorbidity Index (2 ≤ vs. 0 or 1)	21.0 vs. 19.2	1.00 (0.56–1.81)	0.988		
Lauren classification (diffuse or Mixed vs. intestinal)	16.6 vs. 20.3	1.15 (0.75–1.77)	0.507		
Macroscopic type (type 4 vs. other)	12.6 vs. 20.1	1.60 (0.80–3.19)	0.185	2.13 (1.04–4.34)	0.038
HER2 score (3 + vs. 2 +)	18.2 vs. 22.6	1.06 (0.59–1.92)	0.839		
Regimen (Platinum drug ( +) vs. Platinum drug (–))	20.3 vs. 12.0	0.59 (0.35–1.00)	0.049	0.63 (0.37–1.07)	0.089
Number of metastatic sites (2 ≤ vs. 0–1)	13.7 vs. 21.1	1.99 (1.30–3.04)	0.002	2.05 (1.34–3.15)	0.001
Previous chemotherapy (yes vs. no)	14.5 vs. 19.6	1.39 (0.83–2.34)	0.212		
Previous gastrectomy (yes vs. no)	19.2 vs. 20.6	1.07 (0.69–1.67)	0.752	–	
PTEN (loss vs. positive)	13.3 vs. 21.0	1.78 (1.08–2.94)	0.023	1.83 (1.09–3.07)	0.022

*HR* hazard ratio, *95% CI* confidence interval; platinum drug, capecitabine or cisplatin; *PTEN* phosphatase and tensin homolog, *HER2* human epidermal growth factor type 2.

^a^Months.

^b^Cox proportional hazard model.

### Inhibitory effect of Tmab on cell growth is specifically suppressed by PTEN knockdown in HER2-GEA cell lines

**Figure 2:**
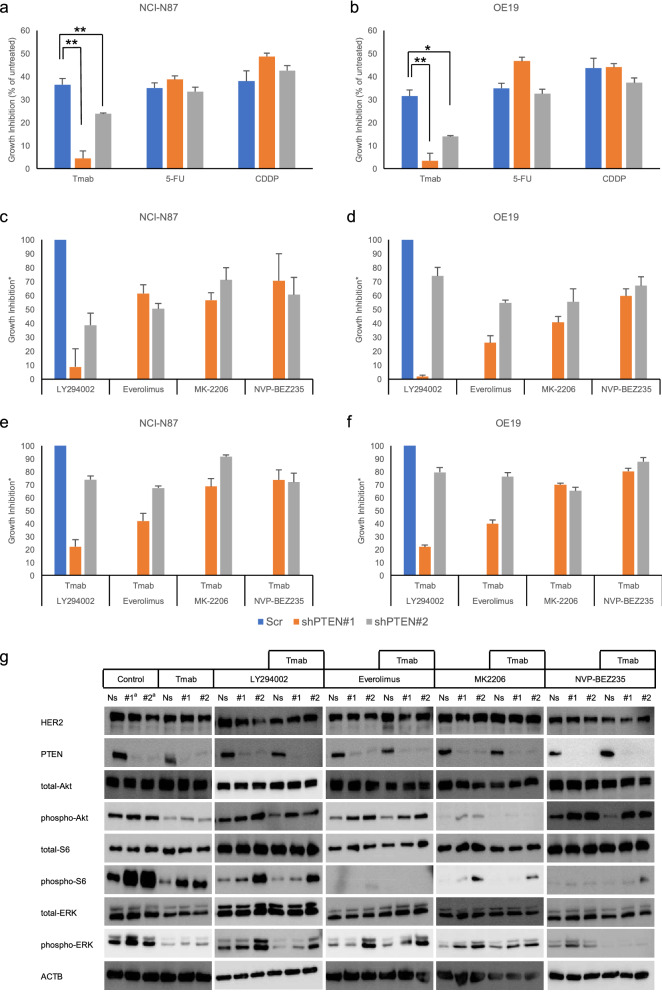
Cell growth inhibition. (**a**) N87 cells treated with Tmab, 5-FU, and CDDP. (**b**) OE19 cells treated with Tmab, 5-FU, and CDDP. (**c**) NCI-N87 cells treated with PI3K inhibitors. (**d**) OE19 cells treated with PI3K inhibitors. (**e**) NCI-N87 cells treated with PI3K inhibitors and Tmab. (**f**) OE19 cells treated with PI3K inhibitors and Tmab. Cell viability was measured using the WST-8 colorimetric assay. PTEN knockdown was performed with shRNA (shPTEN#1 and shPTEN#2). Treatment was performed for 120 h using the following conditions: Tmab (10 μg/mL), 5-FU (1 µM for NCl-N87 cells and 10 µM for OE19 cells), CDDP (1 µM), LY294002 (5 µM for NCl-N87 cells and 10 µM for OE19 cells), everolimus (10 nM), MK-2206 (500 nM for NCl-N87 cells and 5 µM for OE19 cells), and NVP-BEZ235 (50 nM for NCl-N87 cells and 500 nM for OE19 cells). Cell growth inhibition was calculated using the following formula: [1 − experimental absorbance (treated well)/control absorbance (untreated well)] × 100; except for (**a**) and (**b**), all values are expressed as a ratio with the value of the scrambled cells set as 100%. *n* = *3* in each group. (**g**) Influence of PI3K inhibitors on NCl-N87 cells. Western blotting analysis of PI3K and MAPK. PTEN knockdown was achieved using siRNA (siPTEN#1 and siPTEN#2). The treatment duration was 24 h, and the concentrations were the same as above. *p < 0.05, **p < 0.001 using Student’s *t* test. #1^a^ was siPTEN#1, #2^a^ was siPTEN#2.

To investigate the drug sensitivity in relation to the PTEN expression status in HER2-GEA, both NCI-N87 and OE19 cells were used to establish two types of stable PTEN-knockdown clones and a scrambled control clone by lentiviral infection of short hairpin RNAs (shRNAs) (referred to as ‘‘shPTEN#1” and ‘‘shPTEN#2” for each cell line; Supplementary Fig. 1). The cell viability assay showed that the tumor growth inhibition rate of Tmab was significantly decreased in the PTEN knockdown clones of both cell lines (Fig. 2a,b), as demonstrated in a previous study^13^. In contrast, the tumor growth inhibition rates of 5-FU and CDDP did not significantly vary in either cell line with or without PTEN knockdown.

### Molecular-targeted drugs inhibiting downstream factors in the PI3K pathway have an antiproliferative effect in PTEN knockdown HER2-GEA cell lines

To identify effective molecular targets in addition to Tmab treatment for PTEN knockdown in HER2-GEA, we evaluated the growth inhibitory activity of PI3K pathway inhibitors: LY294002, PI3K inhibitor; everolimus, mTORC1 inhibitor; MK-2206, Akt inhibitor; and NVP-BEZ235, potential inhibitor of PI3K, mTORC1, and mTORC2 (Supplementary Fig. 2). LY294002 had a limited growth inhibitory effect in both PTEN knockdown cell lines of NCI-N87 and OE19 with or without Tmab. Growth inhibition by everolimus combined with Tmab was weak in NCI-N87 cells. MK-2206 had a relatively high growth inhibitory effect in both PTEN knockdown lines of NCI-N87 and OE19 with or without Tmab, and the inhibitory effect was augmented in combination with Tmab. The growth inhibitory effect of NVP-BEZ235 with Tmab in both PTEN knockdown NCI-N87 and OE19 cells was more than 70%, compared with that in the control cells (Fig. 2c–f).

To examine the pharmacokinetics of Tmab or PI3K inhibitors, the phosphorylation status of Akt, S6, and extracellular-signal-regulated kinase (ERK), which are critical downstream factors in the PI3K and MAPK pathways, was examined by western blotting (Fig. 2g). All original images, including controls, are presented in the supplementary information. The levels of phospho-Akt, phospho-S6, and phospho-ERK were increased in PTEN knockdown lines of NCI-N87, compared with those in control cells transfected with nonsilencing small interfering RNAs (siRNAs). Tmab alone suppressed the levels of phosho-Akt, phospho-S6, and phospho-ERK, but their levels were increased using Tmab alone in PTEN knockdown cell lines. LY294002 did not affect the levels of phospho-Akt, phospho-S6, and phospho-ERK in PTEN knockdown cells. Everolimus, in combination with Tmab, lowered the phospho-S6 level, but did not affect the levels of phospho-Akt and ERK in PTEN knockdown cells. MK-2206 suppressed the levels of phospho-Akt and phospho-S6 but did not affect the phospho-ERK level in PTEN knockdown cells, even when tested in combination with Tmab. NVP-BEZ235 alone reduced the levels of phospho-Akt and phospho-S6 but did not affect the phospho-ERK level in PTEN knockdown cells. However, the combination of NVP-BEZ235 and Tmab reduced the levels of phospho-Akt, phospho-S6, and phospho-ERK in PTEN knockdown cells.

## Discussion

This multicenter retrospective study demonstrated that PTEN loss is associated with a poor response to Tmab in patients with HER2-GEA. Although this was a retrospective study, the clinical objective evaluation and PTEN immunohistochemistry (IHC) were performed under certain conditions and assessed by multiple physicians and a pathologist who were blinded for the clinical outcome, which makes the results highly reliable. To date, there have been a few studies in which patients with HER2-GEA were divided into two groups according to the therapeutic effect of Tmab, and their histological characteristics and gene expression pattern were evaluated^20–23^. These studies focused on PTEN loss as an important factor of Tmab resistance. To our knowledge, this study is the first to analyze the expression pattern of PTEN in a blind manner to patient outcomes with a central judgment; furthermore, the pathological effect and prognosis of patients were evaluated based on the expression pattern of PTEN. Currently, there is no unified method for assessing PTEN status, and PTEN loss has not been clinically used as a prognostic biomarker in patients with HER2-GEA. As positive aspect of this study is that the assessment of PTEN status is simple and easily applicable to clinical practice. Moreover, we found that combining Tmab with PI3K pathway inhibitors might overcome Tmab resistance in patients with HER2-GEA with PTEN loss. To our knowledge, this is the first study to simultaneously evaluate the cell growth inhibitory effect of various PI3K inhibitors using PTEN knockdown HER2 GEA cell lines. This result suggested the possibility of developing a new therapeutic approach for patients with HER2-GEA.

We previously reported that the continuous activation of HER2 downstream signaling pathways by PTEN loss could be a potential mechanism for lowering the efficacy of Tmab in patients with HER2-GEA^13^. Our study provides guidance for improving personalized medicine in gastric cancer treatment. The PTEN expression status confirmation in patients with HER2-GEA should allow the early identification of patient subsets with effective Tmab treatment response and the advance planning of additional treatment for patient subsets with a less effective Tmab treatment response. In the GRANTE-1 trial, everolimus (mTOR1 inhibitor) failed to show statistically significant efficacy in all patients with gastric cancer, but the study findings revealed that everolimus was clearly effective in a subpopulation^18^. Furthermore, the SWOG phase II trial demonstrated the potential efficacy of Akt inhibitor MK2206 in patients with GEA^24^. Based on the results of our study, MK-2206 may have been effective in patients with PTEN loss HER2 GEA. It is possible that these molecular-targeted drugs are effective in a subpopulation of patients with HER2-GEA identified using biomarkers.

We used the best overall response of each patient according to RECIST v1.1 to evaluate the clinical objective response. Almost all study subjects received Tmab treatment combined with conventional therapy. We retrospectively demonstrated that CDDP and 5-FU had at least a transient and constant effect on HER2-GEA regardless of PTEN expression. The primary endpoint of our study was the DCR, defined as the period during which Tmab suppressed the progression of HER2-GEA. Importantly, we determined that the duration of the best overall response to Tmab-CTx was longer in the PTEN-positive group than in the PTEN-loss group, and this tendency was more pronounced in patients with target lesions. Furthermore, the PTEN-loss group had a shorter SD duration than the PTEN-positive group. These outcomes are consistent with the Tmab mechanism based on blocking cell proliferation by inhibiting intracellular signaling^25,26^.

Our multivariate analysis indicated that PTEN loss can be a predictive factor for significantly shorter PFS and OS periods, indicating that PTEN loss in patients with HER2-GEA was associated with insufficient Tmab treatment response, resulting in shorter OS. Although the ratios of platinum-based and 5-FU-based combinations with Tmab differed significantly between the PTEN-positive and PTEN-loss groups, only the number of patients who received platinum-based treatment was included in the multivariate analysis. This value was selected because most cases from both study groups were treated with 5-FU and platinum-based chemotherapy. Originally, our study included patients who were diagnosed as HER2-positive and treated with Tmab. As important factors such as the number of metastatic sites, macroscopic type, and Lauren classification were also adjusted for confounding, we assumed that our results were sufficiently reliable.

However, it is still unclear whether PTEN loss is an independent prognostic factor for all patients with HER2-GEA because this study did not include patients with HER2-GEA who did not receive Tmab treatment. Moreover, the relationship between PTEN expression and patient prognosis in patients with HER2-negative GEA remains unclear. As a recent study demonstrated that PTEN negative was a poor prognostic factor for all patients with gastric cancer^27^, it might be necessary to consider personalized treatment for PTEN-negative gastric cancer regardless of HER2 expression. Further clinical studies are needed to determine treatment strategies that are required for patients with PTEN-negative GEA.

Our in vitro experiments suggested that combining Tmab with BEZ235 (dual inhibitor of PI3K and mTOR) was more effective in patients with HER2-GEA with PTEN loss than combining Tmab with other PI3K pathway inhibitors. Under the PTEN loss status, the PI3K pathway is activated downstream of HER2 by a complex feedback mechanism; thus, the suppression of a single factor may not be sufficient for growth suppression of HER2-GEA. Interestingly, BEZ235 with Tmab inhibited the expression of pERK, which is a key downstream factor of the MAPK pathway. As described in previous reports, complex interactions between the PI3K and MAPK pathways make it difficult to explain why a PI3K/mTOR inhibitor affects the expression of pERK^28^. However, our results implied that the concurrent inhibition of upstream and downstream factors of the PTEN action site could effectively inhibit the growth of HER2-GEA. Dual PI3K-mTOR inhibitors have been investigated as new therapeutic options^29^, but the risk of adverse effects should be assessed for future clinical applications.

There are several causes of poor response to Tmab other than PTEN loss in patients with breast cancer, which may be due to dimerization of HER2 and other EGFR receptors such as HER3^15^. HER3, frequently overexpressed in high-grade dysplastic lesions of the gastroesophageal junction, is a marker of invasive progression^30^, this receptor should be considered more than HER2 heterodimerization partner^31^. Therefore, the selection of PI3K inhibitors in patients with PTEN loss HER2-GEA should be considered, including its expression profile.

The current study had several limitations. Firstly, as this was a retrospective study, we could not rule out the effects of unmeasured confounders and case selection bias. For example, there were differences in the combination regimens with Tmab, as well as second- or third-line therapies, including empirical treatment. Secondly, as we conducted a multicenter retrospective study, the follow-up interval and the timing of the following imaging study depended on each attending doctor. However, most of the patients were subjected to a diagnostic imaging examination within 3 months from Tmab-CTx initiation, and the physiological or image analysis findings for each patient were reviewed by two independent physicians, which likely made these assessments sufficiently reliable. Thirdly, the expression pattern of HER2 and PTEN in patients with gastric cancer is potentially heterogeneous^32–36^, and IHC of HER2 and PTEN can only be confirmed by biopsy or surgical specimens. Thus, it is unclear how HER2 and PTEN are expressed when the disease progresses or when there is recurrence. Establishing a treatment strategy for recurrence with multidrug resistance will remain a critical issue in gastric cancer therapy.

Our retrospective study revealed that a considerable subset of HER2-GEA patients with PTEN loss is potentially resistant to Tmab and could benefit from additional molecular-targeted therapies suppressing the activation of the PI3K pathway. Our study could provide guidance for overcoming a terrible GEA outcome by pioneering precision medicine with molecular-targeted therapy.

## Methods

### Patients, clinical data, and pathological tissue collection

This study was approved by the review board of each hospital, including Kyoto University (Confirmation No. R1230), and patients provided informed consent for sample use and data analysis through opt-out agreements. This multicenter, retrospective observational study was conducted at Kyoto University, Osaka Red Cross Hospital, Kyoto Medical Center, Kyoto Katsura Hospital, Tenri Hospital, and Otsu Municipal Hospital. The study protocol was approved by the ethics committee of Kyoto University and each hospital. This study was conducted in accordance with the Ethical Guidelines for Medical and Health Research Involving Human Subjects and the Declaration of Helsinki.

We retrospectively enrolled 174 patients who received systemic Tmab-CTx for HER2-GEA between January 2011 and December 2016 in any of the participating hospitals. Clinical data and pathological tissue samples were collected from patients in each hospital. We included patients (1) whose HER2 score was 2 plus and the fluorescence in situ hybridization result was positive or whose HER2 score was 3 plus, and (2) who had received Tmab-CTx. The exclusion criteria were as follows: (1) pathological tissue samples or detailed clinical information could not be obtained, and (2) could not be evaluated for the objective response to chemotherapy. From the database of each hospital, we obtained the clinical information of these patients as they received Tmab-CTx retrospectively. Comorbidity was assessed using the CCI.

Pathological tissue samples with formalin-fixed, paraffin-embedded sections were obtained from the specimens of surgeries or endoscopic biopsy when the case was diagnosed as HER2-GEA. These samples were processed in the pathological department of each hospital.

### PTEN expression analysis

Tissue sections were analyzed for PTEN by IHC with PTEN monoclonal antibody (clone 138G6, #9559, Cell Signaling Technology, Danvers, MA; diluted 1:200) at Kyoto University, as reported previously^13,37^. PTEN IHC was subjectively scored as absent (0) without detectable immunostaining in the whole cancer specimen, as weak (1+) with low cytoplasmic staining, as moderate (2+) with intermediate staining (between weak and strong), and as strong (3+) with intense staining (Supplementary Fig. 3). PTEN loss was defined as negative staining (score 0) of cells in more than 75% of the tumor^13^. The results were interpreted by three independent observers, including a specialist of pathology, and discrepancies were discussed to obtain a final result.

### Clinical response to Tmab-CTx

We evaluated the clinical objective response to Tmab-CTx according to RECIST version 1.1^19^. The best overall response to Tmab-CTx was recorded as the objective response of each patient. This evaluation was based on radiological images, including computed tomography results, along with magnetic resonance imaging and relevant physical examination findings, all of which were reviewed retrospectively in a blinded manner by at least two clinicians.

The sum of the proportion of complete response (CR) and partial response (PR) represented the response rate used to compare the PTEN-loss group with the PTEN-positive group. DCR, which was defined as the sum of the proportions of CR, PR, SD, and non-CR/non-PD, was used as the study’s primary endpoint for comparing these two groups. In addition, DCR was evaluated in patients with target lesions. The duration of SD, which represented the period from the Tmab-CTx initiation date to the progressive disease (PD) diagnosis date, was also evaluated and compared between the groups.

### OS and PFS

OS was defined as the period from the date of Tmab-CTx initiation to the date of the patient’s death. PFS was defined as the period from the date of Tmab-CTx initiation to the date of disease “progression” onset based on an objective evaluation of the patient chart or to the date of patient’s death.

### Cell lines, cell culture, and reagents

Human gastric adenocarcinoma cell line NCI-N87 (RRID: CVCL_1603) was purchased from American Type Culture Collection (Manassas, VA). Human esophageal adenocarcinoma cell line OE19 (RRID: CVCL_1622) was purchased from European Collection of Cell Cultures (Salisbury, UK). These cell lines were confirmed by STR profiling and were Mycoplasma free. Cell lines were cultured in RPMI 1640 medium supplemented with 10% fetal bovine serum, penicillin (100 U/mL), and streptomycin (100 μg/mL; Life Technologies) and were incubated at 37 °C in a humidified chamber containing 5% CO_2_. Tmab was provided by Chugai Pharmaceutical (Tokyo, Japan) for nonclinical investigations; 5-FU was purchased from Wako (Tokyo, Japan); CDDP was provided by YAKULT HONSHA (Tokyo, Japan). LY294002 and NVP-BEZ235 were purchased from Cayman Chemical (Ann Arbor, MA). Everolimus (RAD001) and MK2206 were purchased from Selleck Chemicals (Houston, TX).

### Small interfering RNA and short hairpin RNA

Two distinct siRNA species targeting PTEN (siPTEN #1, Hs_PTEN_6 FlexiTube siRNA, SI00301504; siPTEN #2, Hs_PTEN_8 FlexiTube siRNA, SI03048178) and nonsilencing control siRNA (AllStars negative control siRNA, SI03650318) were purchased from Qiagen and transfected with Lipofectamine RNAiMAX (Invitrogen Life Technologies, Carlsbad, CA) according to the manufacturer’s protocol.

The shRNA vector (PTEN Human shRNA Plasmid Kit, TL320498) was purchased from Origene (Rockville, MA). The plasmid kit contained a non-effective 29-mer scrambled shRNA and four unique 29-mer shRNA constructs, #A, #B, #C, and #D, in the lentiviral GFP vector, from which we selected #C and #D shRNA because they had better PTEN knockdown efficacy. The packaging vector (psPAX2, plasmid 12260) and envelope vector (pMD2.G, plasmid 12259) were purchased from Addgene (Cambridge, MA). The shRNA lentivirus containing shRNA constructs were prepared according to the manufacturer’s protocol, and the cell lines were infected with them. We selected stable knockdown clones by flow cytometry to collect GFP-positive cells.

### Western blotting

Cells were lysed in sodium dodecyl sulfate lysis buffer supplemented with a protease inhibitor cocktail (Nacalai Tesque, Kyoto, Japan) and phosphatase inhibitor cocktail (Nacalai Tesque). A total of 20 μg of whole-cell lysate was subjected to sodium dodecyl sulfate-polyacrylamide gel electrophoresis and transferred on to a polyvinylidene difluoride membrane (Merck Millipore). Membranes were probed with specific primary antibodies against HER2 (polyclonal, #2242), PTEN (clone 138G6, #9559), pan-Akt (clone C67E7, #4691), phosphorylated Akt (Ser473, clone D9E, #4060), p44/p42 mitogen-activated protein kinase [MAPK; ERK1/2] (clone 137F5, #4695), phosphorylated p44/p42 MAPK (ERK1/2) (Thr202/Tyr204, clone D13.14.4E, #4370), S6 ribosomal protein (clone 5G10, #2217), phosphorylated S6 ribosomal protein (Ser235/236, clone D57.2.2E, #4858) (Cell Signaling Technology), and horseradish peroxidase (HRP)-conjugated secondary antibody (Dako, Santa Clara, CA). HRP-conjugated β-actin antibody (Sigma-Aldrich, St. Louis, MO) was used as a loading control. Bands were visualized using a Pierce Western blotting substrate kit (Thermo Scientific).

### Cell viability and cell growth inhibition assay

Cell viability was measured using the WST-8 colorimetric assay with the Cell Counting Kit-8 (CCK-8; Dojindo, Kumamoto, Japan). Exponentially growing cells (2500–3500/100 μL/well) were seeded in triplicate into 96-well plates and cultured in reagent-containing medium for 120 h. Thereafter, 10 μL of CCK-8 was added to each well, and incubation was continued for 3 h. The absorbance of the solution at 450 nm was measured using a GloMax-Multi detection system (Promega, Madison, WI) to calculate the number of viable cells per well. The working concentrations of the reagents were in the range at which the tumor growth inhibition rate was 30–50%. We investigated the tumor inhibition rate of each reagent in the original cell lines at various concentrations (Supplementary Fig. 4). The optimized reagent concentrations in NCl-N87 and OE19 cell cultures were derived from preliminary experiments: Tmab, 10 µg/mL in both cell lines; 5-FU, 1 and 10 µM in NCl-N87 and OE19, respectively; CDDP, 1 µM in both cell lines; LY294002, 5 and 10 µM in NCl-N87 and OE19, respectively; everolimus, 10 nM in both cell lines; MK-2206, 500 nM and 5 µM in NCl-N87 and OE19, respectively; NVP-BEZ235, 50 and 500 nM in NCl-N87 and OE19, respectively.

### Statistical analysis

All values are expressed as mean ± standard deviation. All in vitro experiments were repeated at least three times. Categorical data were analyzed using Fisher’s exact test. Continuous variables were analyzed using Student’s *t *test. To investigate factors associated with OS or PFS, the multivariate analysis by Cox proportional hazard model was performed for factors that were included in the model with p < 0.20 in the univariate analysis. Survival curves were obtained using to the Kaplan–Meier method and analyzed using the log-rank test. All analyses were two-sided, and differences with p < 0.05 were considered statistically significant. Statistical analyses were performed using JUMP software, version 14.0, Statistical Discovery (SAS, Cary, NC).

## Supplementary Information

Supplementary Figures.

## Data Availability

The data that support the findings of our study are available upon reasonable request from the corresponding author.
