# Author Correction: PTEN is a predictive biomarker of trastuzumab resistance and prognostic factor in HER2-overexpressing gastroesophageal adenocarcinoma

**DOI:** 10.1038/s41598-021-00915-1

**Published:** 2021-10-22

**Authors:** Daiju Yokoyama, Shigeo Hisamori, Yasunori Deguchi, Tatsuto Nishigori, Hiroshi Okabe, Seiichiro Kanaya, Dai Manaka, Yoshio Kadokawa, Hiroaki Hata, Sachiko Minamiguchi, Shigeru Tsunoda, Kazutaka Obama, Yoshiharu Sakai

**Affiliations:** 1 Department of Surgery, Graduate School of Medicine, Kyoto University, 54, Shogoin-Kawahara-cho, Sakyo-ku, Kyoto, 606-8507 Japan; 2 Department of Surgery, Ijinkai Takeda General Hospital, 28-1, Ishida Moriminami-cho, Fushimi-ku, Kyoto, 601-1495 Japan; 3 Department of Gastroenterological Surgery, New Tokyo Hospital, 1271, Wanagaya, Matsudo, Chiba 270-2232 Japan; 4 Department of Surgery, Osaka Red Cross Hospital, 5-30, Fudegasaki-cho, Tennoji-ku, Osaka, 543-8555 Japan; 5 Department of Surgery, Kyoto Katsura Hospital, 17, Yamada-Hirao-cho, Nishikyo-ku, Kyoto, 615-8256 Japan; 6 Department of Gastrointestinal Surgery, Tenri Hospital, 200, Mishima-cho, Tenri, Nara 632-8552 Japan; 7 Department of Surgery, National Hospital Organization Kyoto Medical Center, 1-1, Fukakusa-Mukaihata-cho, Fushimi-ku, Kyoto, 612-8555 Japan; 8 Department of Diagnostic Pathology, Graduate School of Medicine, Kyoto University, 54, Shogoin-Kawahara-cho, Sakyo-ku, Kyoto, 606-8507 Japan

Correction to: *Scientific Reports* 10.1038/s41598-021-88331-3, published online 27 April 2021

The original version of this Article contained an error in the results of the univariate analysis. As the result, in the Methods, under the subheading ‘Statistical analysis’,

“To investigate factors associated with OS or PFS, the multivariate logistic regression analysis was used for factors that were included in the model with p < 0.20 in the univariate analysis.”

now reads:

“To investigate factors associated with OS or PFS, the multivariate analysis by Cox proportional hazard model was performed for factors that were included in the model with p < 0.20 in the univariate analysis.”

Additionally, in Table 2(A), column ‘Variables’

“Number of metastatic lesion”

now reads:

“Number of metastatic sites”

Similarly, in Table 4(A) and (B), first column,

“Number of metastatic lesion (2 ≤ vs. 0–1)”

now reads:

“Number of metastatic sites (2 ≤ vs. 0–1)”

And, in Table 4 legend,

“^b^Logistic regression model”

now reads:

“^b^Cox proportional hazard model”

In the Results, under the subheading ‘PTEN loss is associated with a poor clinical response to Tmab-CTx in patients with HER2-GEA’,

“The proportion of patients who received previous chemotherapy did not significantly vary, but the PTEN-loss group had a significantly lower proportion of patients who underwent gastrectomy than the PTEN-positive group (66% vs. 22%, p < 0.001).”

now reads:

“The proportion of patients who received previous chemotherapy did not significantly vary, but the PTEN-positive group had a significantly lower proportion of patients who underwent gastrectomy than the PTEN-loss group (66% vs. 22%, p < 0.001).”

And, under the subheading ‘PTEN loss has prognostic significance and is a predictive factor for shorter OS and PFS in patients with HER2-GEA receiving Tmab-CTx’,

“We found that two or more lesions of metastases and PTEN loss were significantly related to shorter PFS in patients with HER2-GEA based on the univariate analysis (p = 0.003 and p = 0.020, respectively) and multivariate analysis (p = 0.002 and p = 0.035, respectively). For OS, platinum-containing regimen, two or more lesions of metastases, and PTEN loss were significantly related to shorter OS in patients with HER2-GEA based on the univariate analysis (p = 0.049, p = 0.002, and p = 0.023, respectively). In the multivariate analysis, macroscopic type 4, two or more lesions, and PTEN loss were significantly related to shorter OS (p = 0.038, 0.001, and 0.022, respectively).”

now reads:

“We found that two or more sites of metastases and PTEN loss were significantly related to shorter PFS in patients with HER2-GEA based on the univariate analysis (p = 0.003 and p = 0.020, respectively) and multivariate analysis (p = 0.002 and p = 0.035, respectively). For OS, non-platinum containing regimen, two or more sites of metastases, and PTEN loss were significantly related to shorter OS in patients with HER2-GEA based on the univariate analysis (p = 0.049, p = 0.002, and p = 0.023, respectively). In the multivariate analysis, macroscopic type 4, two or more sites of metastases, and PTEN loss were significantly related to shorter OS (p = 0.038, 0.001, and 0.022, respectively).”

Finally, in the Acknowledgements,

“The authors would like to express sincere gratitude to all members of the KEGG consortium for providing the necessary data and for their cooperation in the critical discussion in carrying out this research. Then, the authors are deeply grateful to Prof. Dr. Masakazu Toi for providing helpful comments and thankful to Daisuke Nishizaki for providing critical comments regarding statistical analyses and review, and Fumie Uemura for technical assistance.”

now reads:

“The authors would like to express sincere gratitude to all members of the KEGG consortium for providing the necessary data and for their cooperation in the critical discussion in carrying out this research. Then, the authors are deeply grateful to Prof. Dr. Manabu Muto and Prof. Dr. Hiroshi Seno for valuable samples and providing helpful comments. Moreover, the authors are deeply thankful to Hiroyasu Abe and Daisuke Nishizaki for providing critical comments regarding statistical analyses and review. Finally, the authors would like to express their deepest gratitude to Prof. Dr. Masakazu Toi for useful comments and Fumie Uemura for technical assistance.”

The original Article has been corrected.

