# Supplementary material for: PTEN is a predictive biomarker of trastuzumab resistance and prognostic factor in HER2-overexpressing gastroesophageal adenocarcinoma
Source: Sci Rep. 2021 Apr 27;11:9013. doi: 10.1038/s41598-021-88331-3 (PMC8079403; doi:10.1038/s41598-021-88331-3)

**PTEN is a predictive biomarker of trastuzumab resistance and prognostic factor in HER2-overexpressing gastroesophageal adenocarcinoma**

Daiju Yokoyama^a^, Shigeo Hisamori^a^*, Yasunori Deguchi^a,b^, Tatsuto Nishigori^a^, Hiroshi Okabe^c^, Seiichiro Kanaya^d^, Dai Manaka^e^, Yoshio Kadokawa^f^, Hiroaki Hata^g^, Sachiko Minamiguchi^h^, Shigeru Tsunoda^a^, Kazutaka Obama^a^, Yoshiharu Sakai^a,d^ on behalf of Kyoto Esophageal and Gastric Surgery Study Group (KEGG)

^a^Department of Surgery, Graduate School of Medicine, Kyoto University

^b^Department of Surgery, Takeda General Hospital

^c^Department of Gastroenterological Surgery, New Tokyo Hospital

^d^Department of Surgery, Osaka Red Cross Hospital

^e^Department of Surgery, Kyoto Katsura Hospital

^f^Department of Gastrointestinal Surgery, Tenri Hospital

^g^Department of Surgery, National Hospital Organization Kyoto Medical Center

^h^Department of Diagnostic Pathology, Graduate School of Medicine, Kyoto University

*Corresponding Author: Shigeo Hisamori*

**Supplementary Data**

Supplementary Figure 1


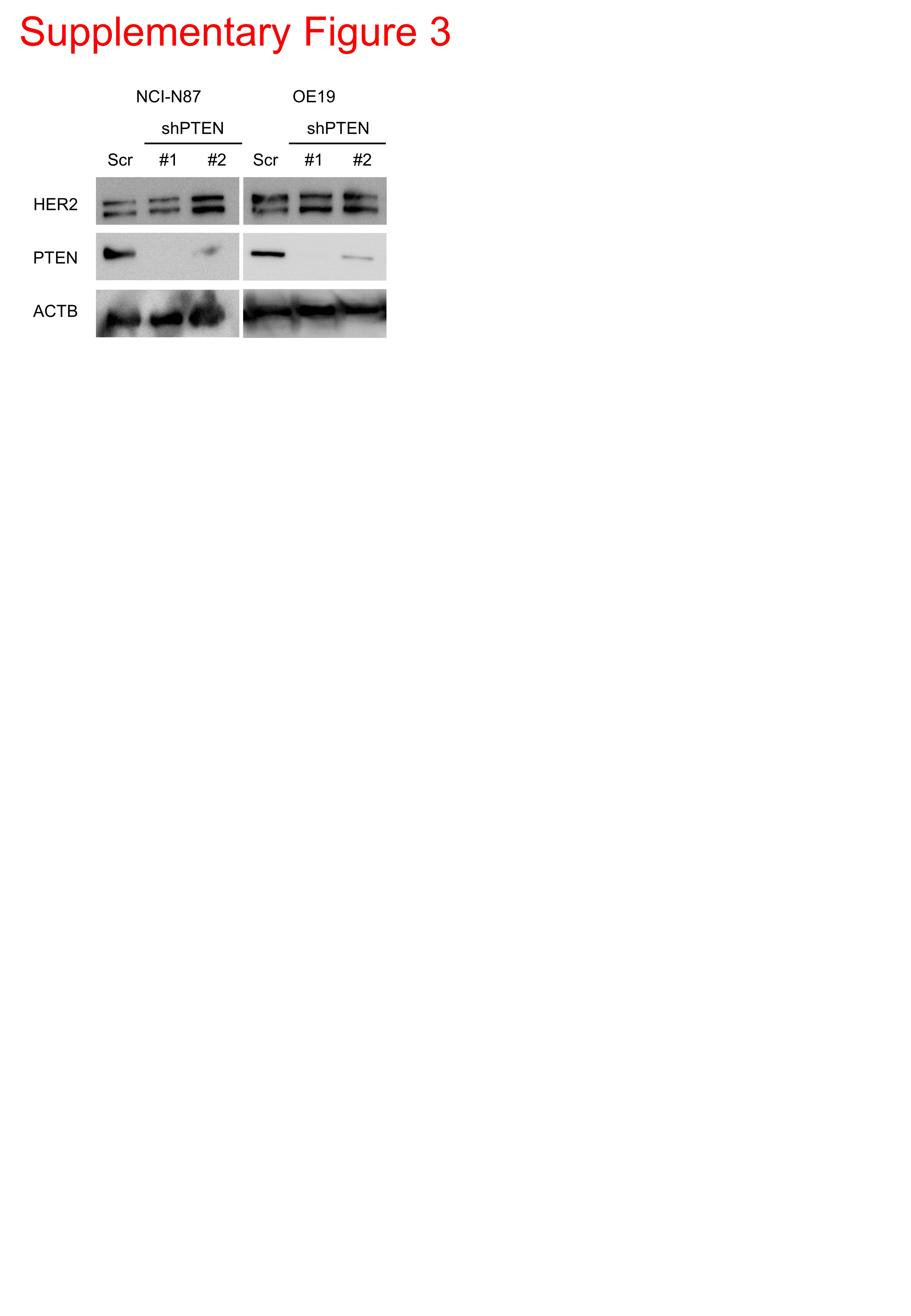


Western blotting analysis of HER2 and PTEN in NCl-N87 and OE19 cells. PTEN knockdown was achieved with shRNA (shPTEN#1 and shPTEN#2). ShPTEN#1 had a better knockdown efficacy than shPTEN#2. Abbreviation: ACTB, actin beta.

Supplementary Figure 2


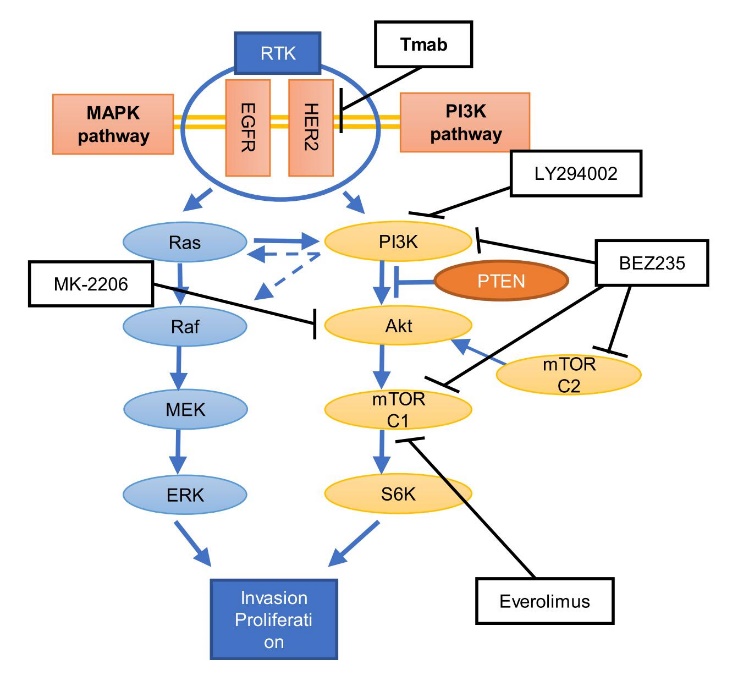


Complex interactions between the PI3K and MAPK pathways: LY294002 inhibits PI3K; everolimus inhibits mTORC1; MK-2206 inhibits Akt; and NVP-BEZ235 potentially inhibits PI3K, mTORC1, and mTORC2.

Supplementary Figure 3


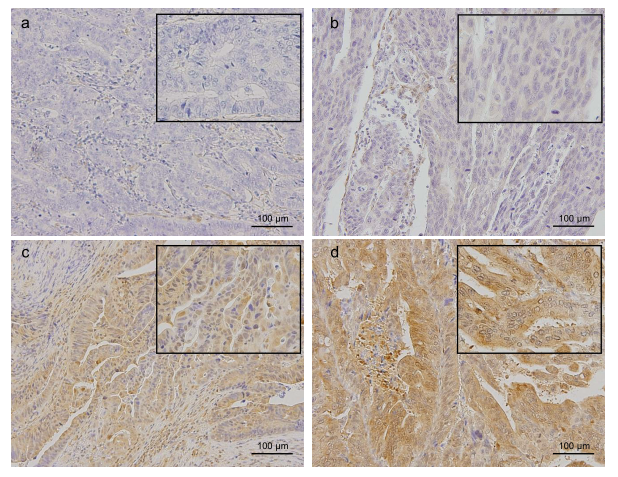


PTEN expression in gastroesophageal adenocarcinoma specimens. Representative PTEN scoring by IHC. (**a)** Score 0: no immunostaining is detectable in the tumor cells. (**b)** Score 1: weak immunostaining. (**c**) Score 2: intermediate immunostaining. (**d**) Score 3: strong immunostaining. PTEN loss was defined as negative staining (score 0) of cells in more than 75% of the tumor. Scale bars represent 100 μm.

Supplementary Figure 4


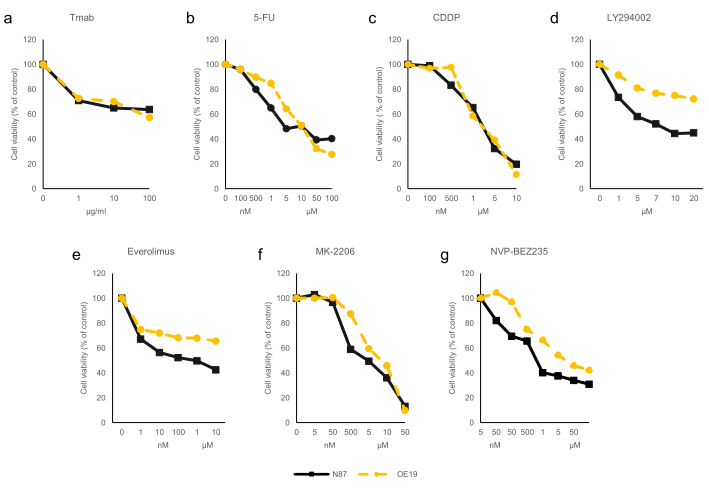


In the cell viability assay, NCI-N87 and OE19 cells were exposed to increasing concentrations of test reagents. Cell viability was measured using the WST-8 colorimetric assay: (**a**) Tmab; (**b**) 5-FU; (**c**) CDDP; (**d**) LY294002; (**e**) Everolimus; (**f**) MK-2206; (**g**) NVP-BEZ235. 5-FU, LY294002, everolimus, MK-2206, and NVP-BEZ235 were dissolved in dimethyl sulfoxide at concentrations of less than 0.1% concentration. Each test reagent concentration was increased until cell viability decreased less than 50%. Each control included DMSO at the same concentration. LY294002 did not inhibit OE19 cell growth over 50%. The reagent exposure time was 120 h. *n = 3* for each test reagent and control.

Original western blot images for Figure 2

These images are from the experiments performed in the order shown below; from the left, control [Ns, siPTEN#1, siPTEN#2], Tmab [Ns, siPTEN#1, siPTEN#2], target agent [Ns, siPTEN#1, siPTEN#2], agent plus Tmab [Ns, siPTEN#1, siPTEN#2]

**Control-Tmab**

HER2


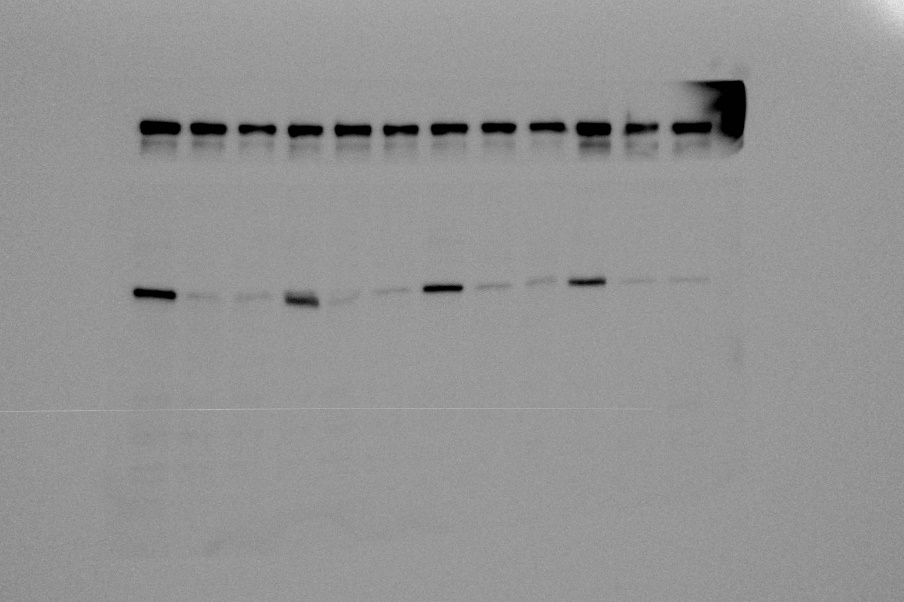


185 kDa

PTEN


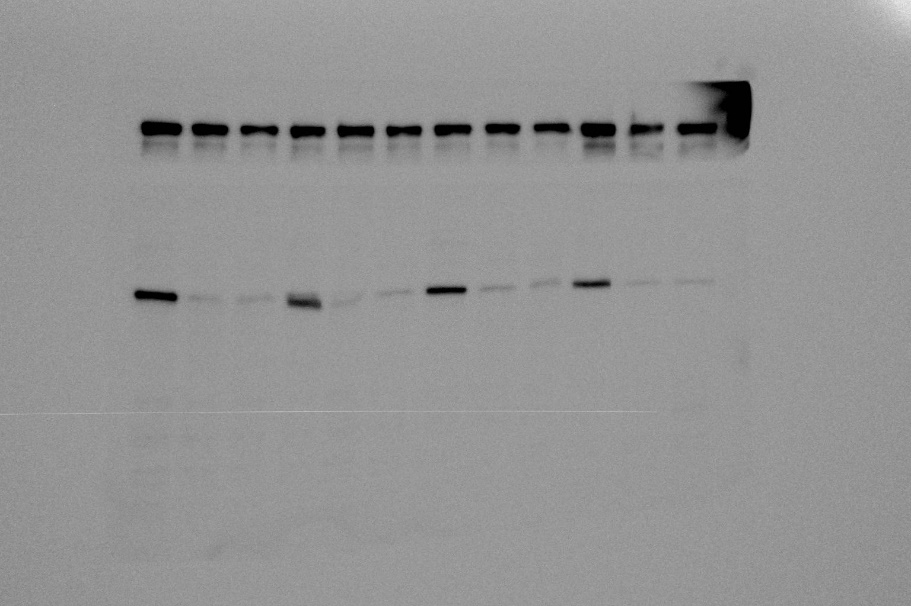


54 kDa

total-Akt


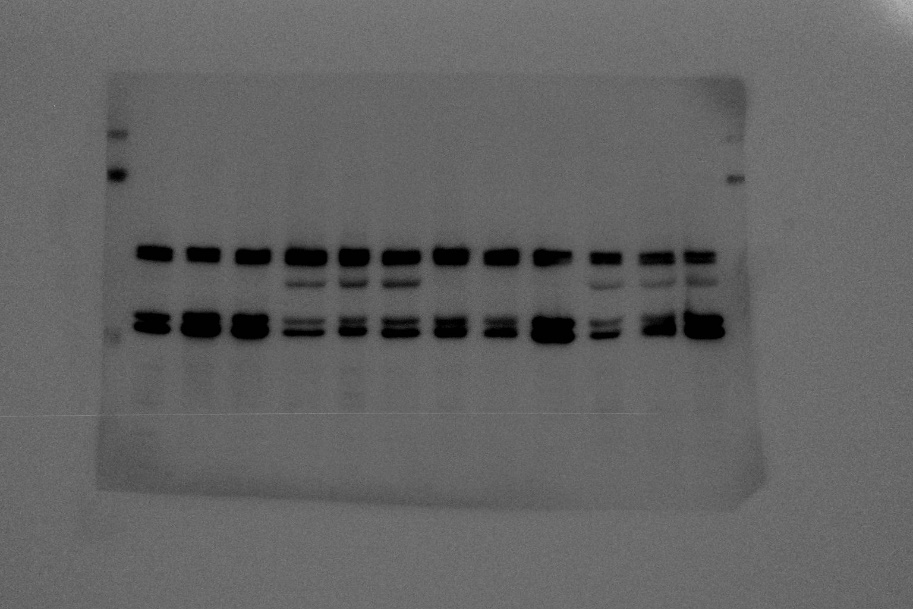


60 kDa

phospho-Akt


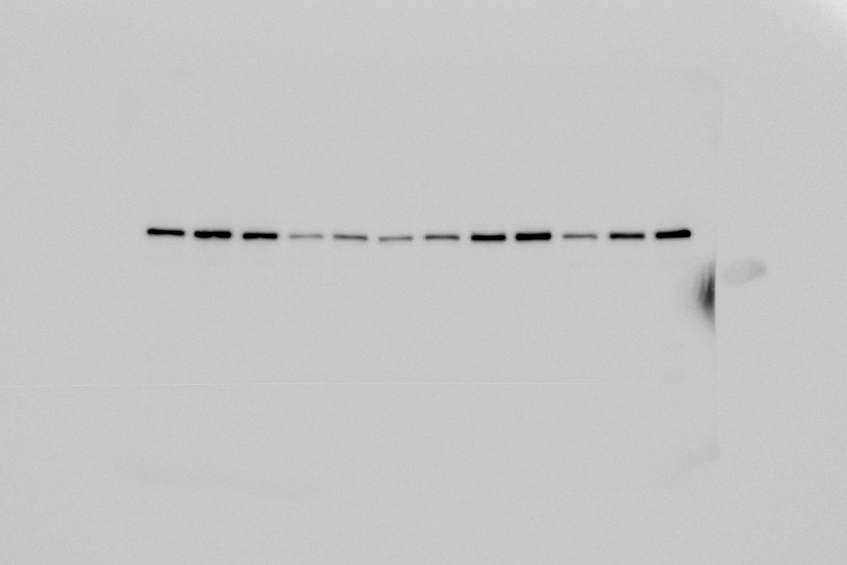


60 kDa

total-S6


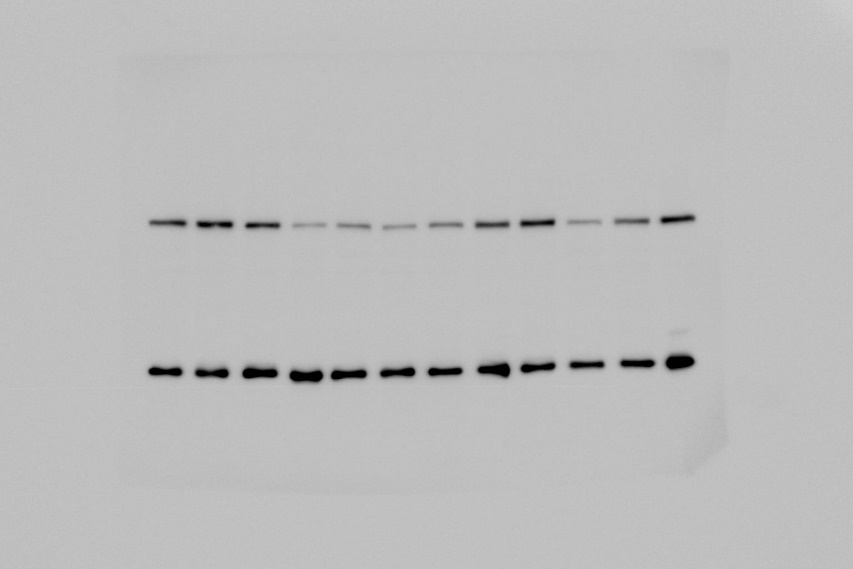


32 kDa

phospho-S6


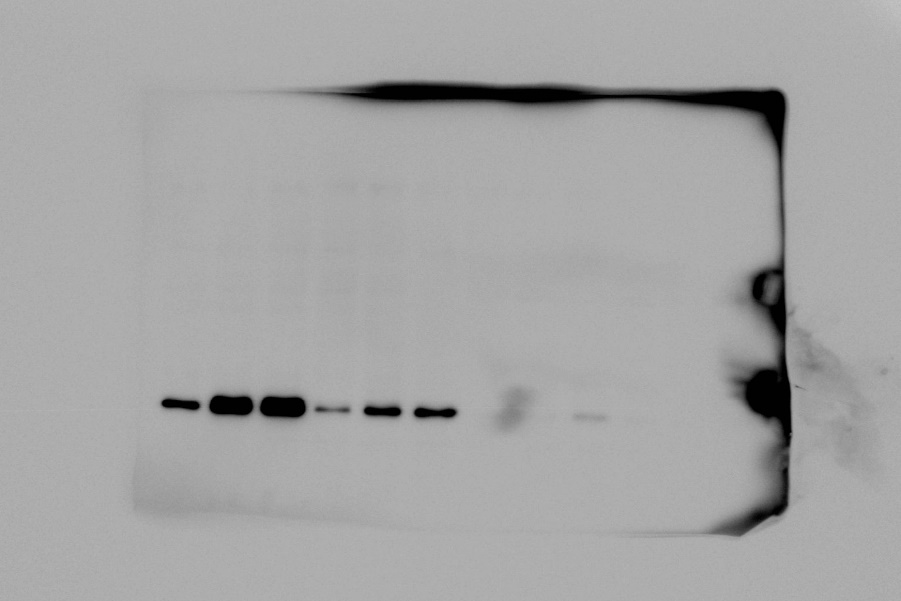


32 kDa

total-ERK


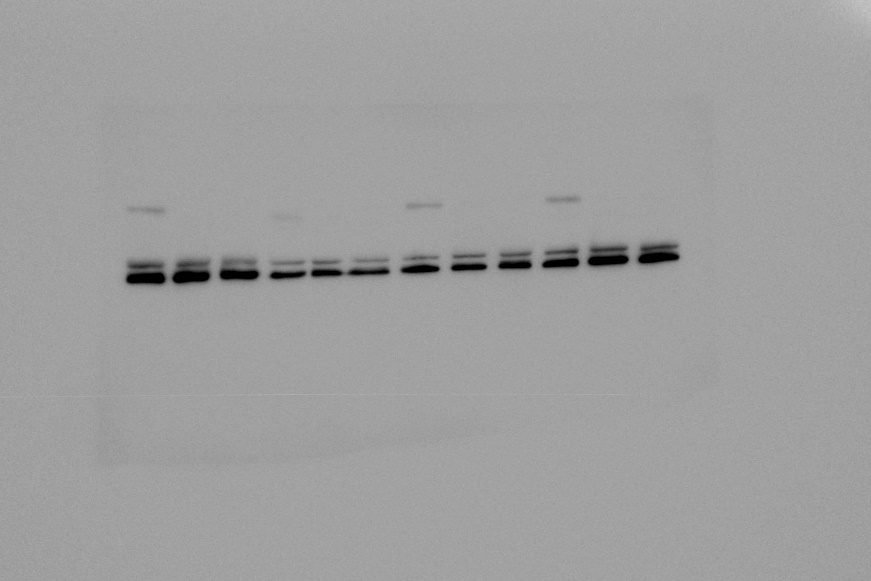


44, 42 kDa

phospho-ERK


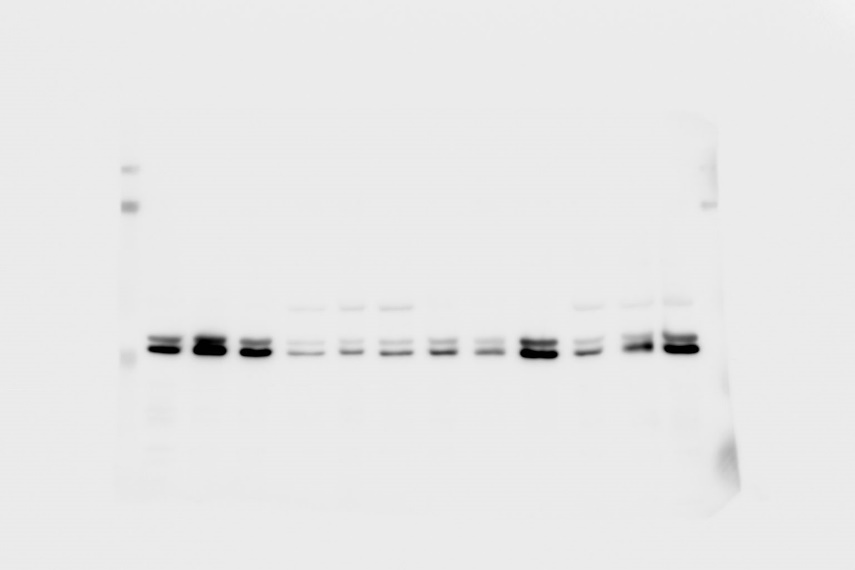


44, 42 kDa

ACTB


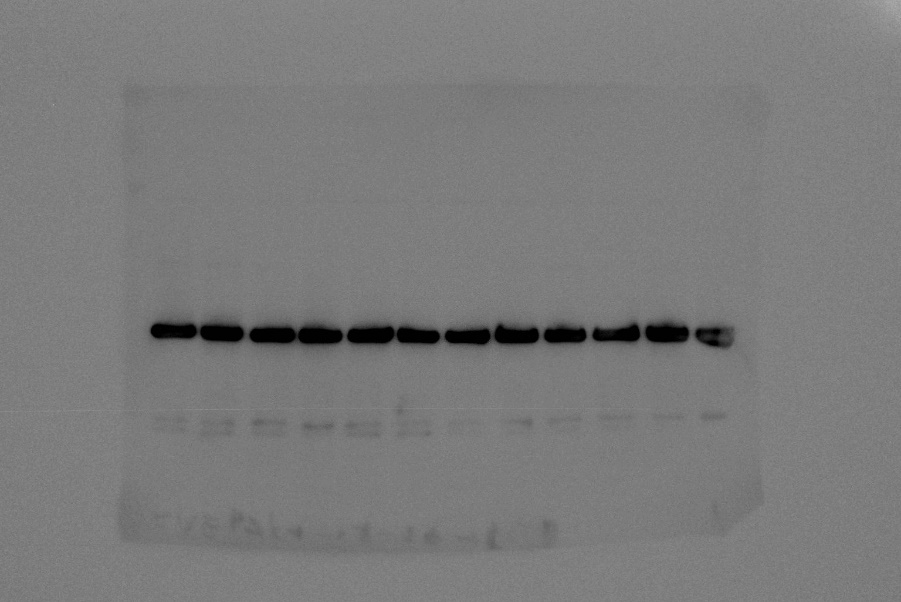


**LY294002**

HER2


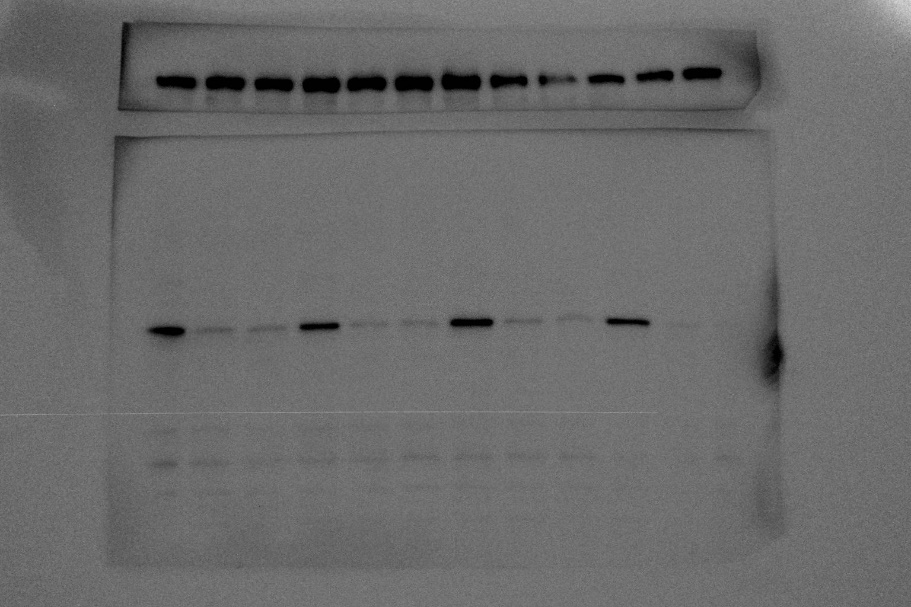


185 kDa

PTEN


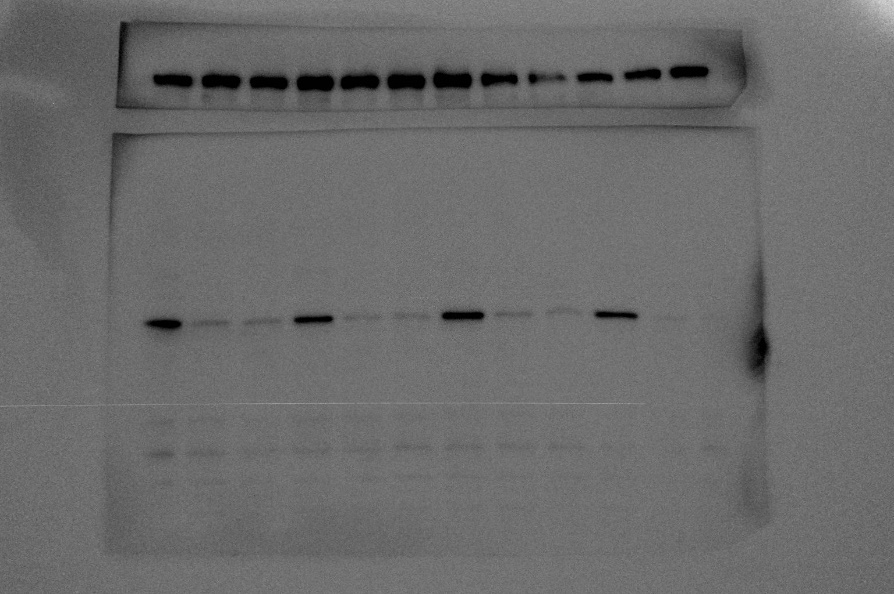


54 kDa

total-Akt
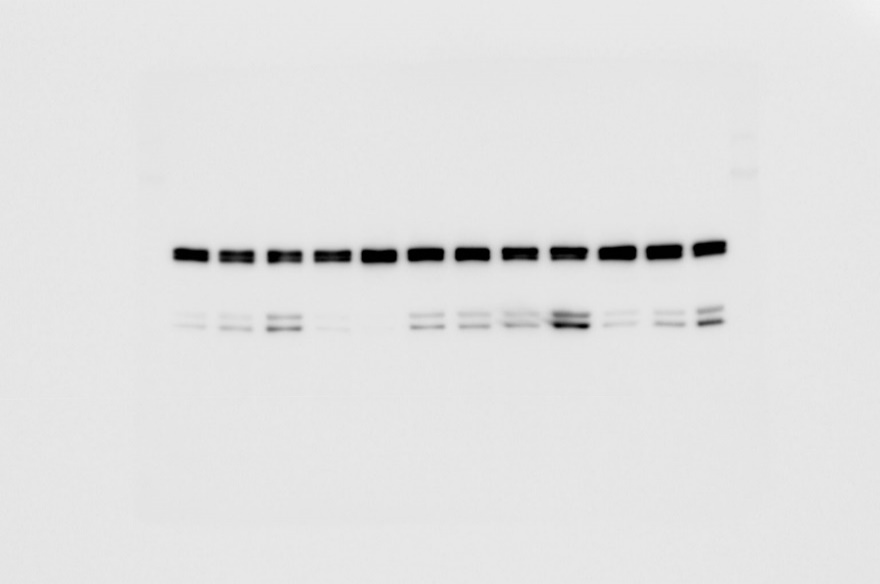


60 kDa

phospho-Akt
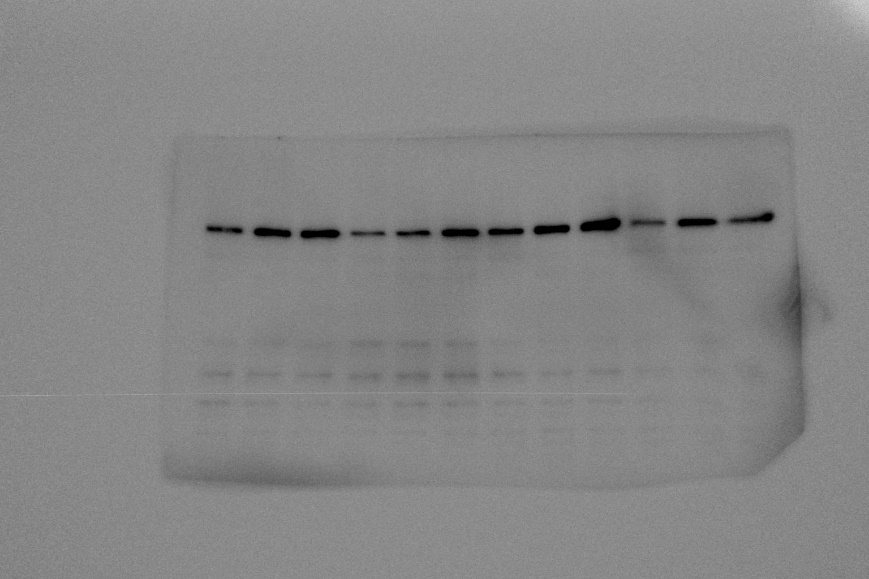


60 kDa

total-S6
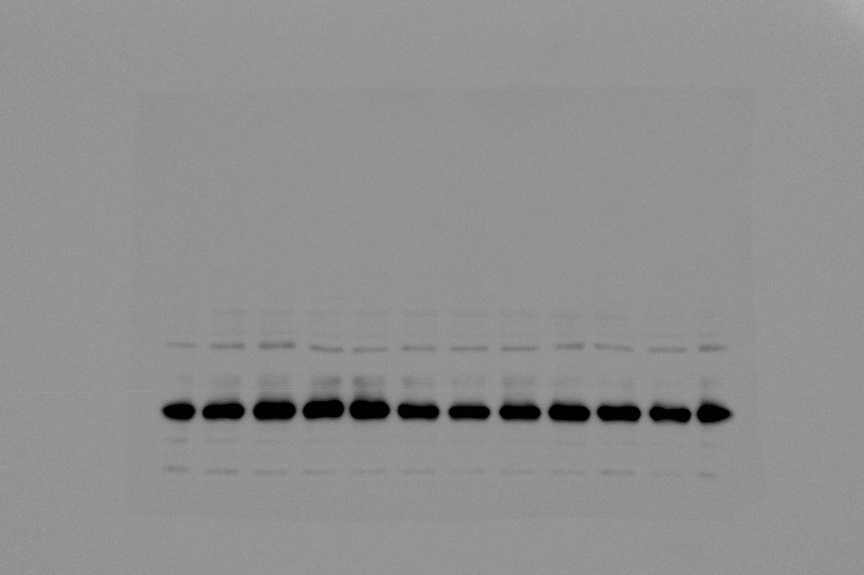


32 kDa

phospho-S6
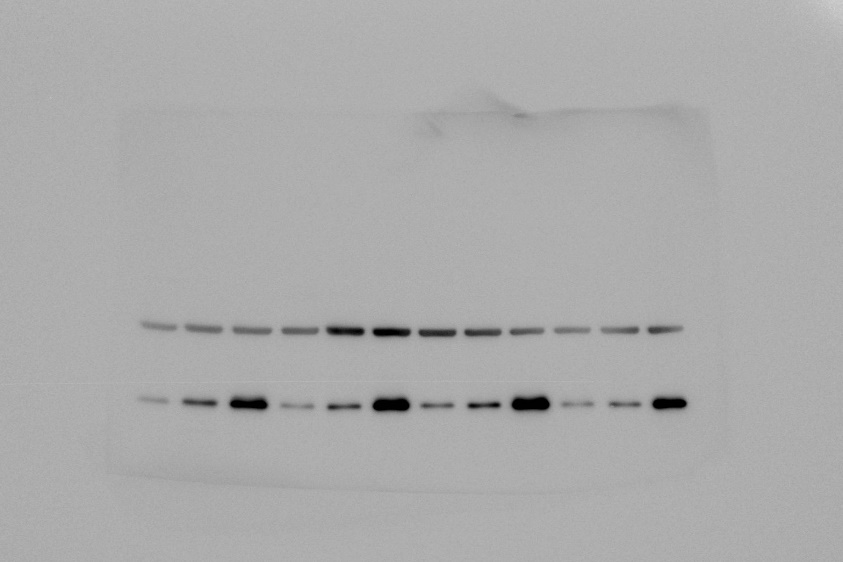


32 kDa

total-ERK
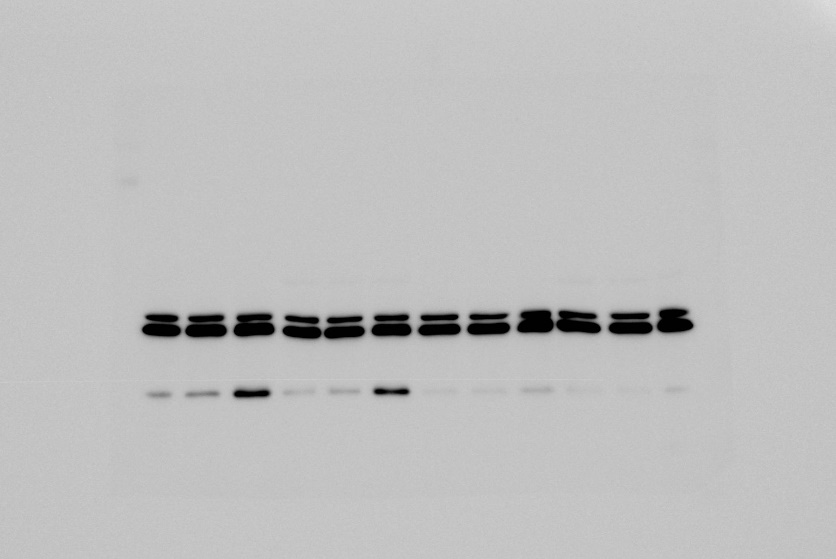


44, 42 kDa

phospho-ERK
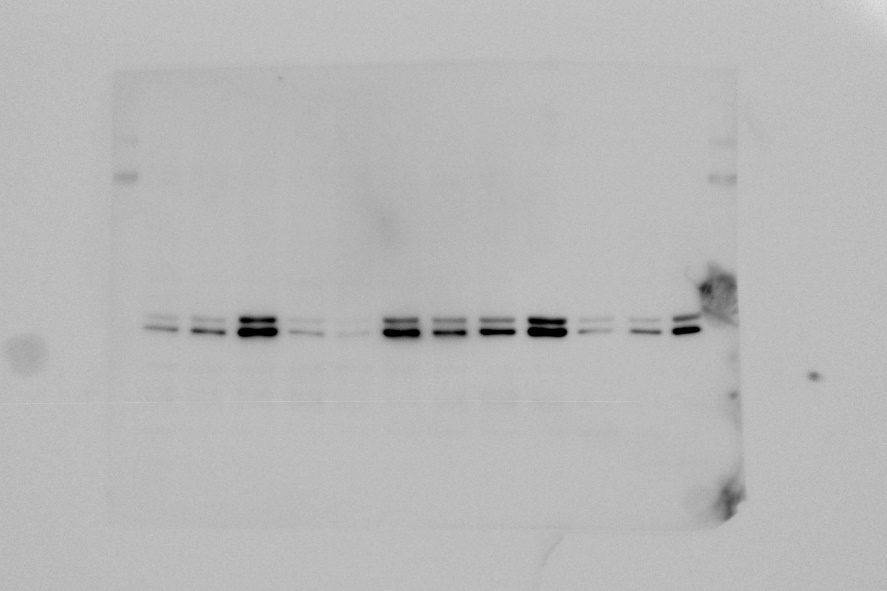


44, 42 kDa

ACTB


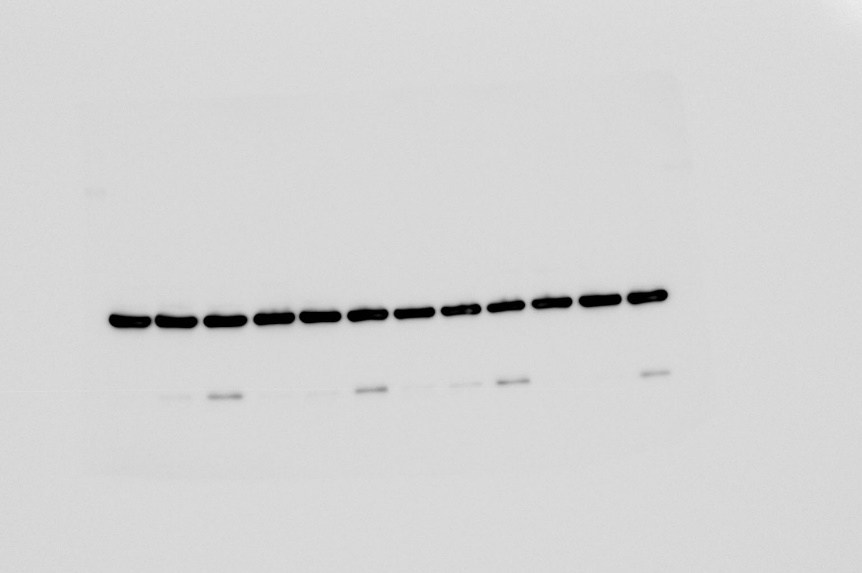


**Everolimus**

HER2


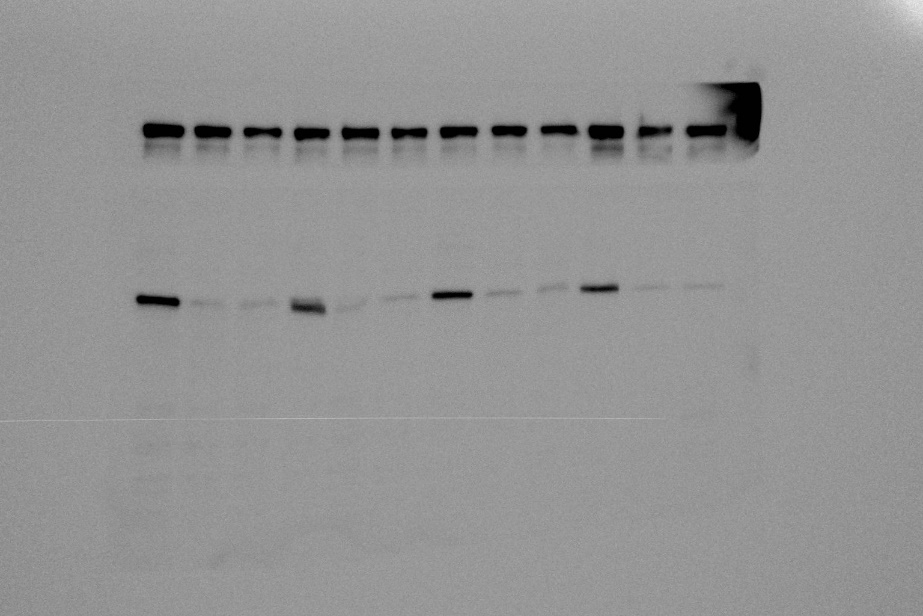


185 kDa

PTEN


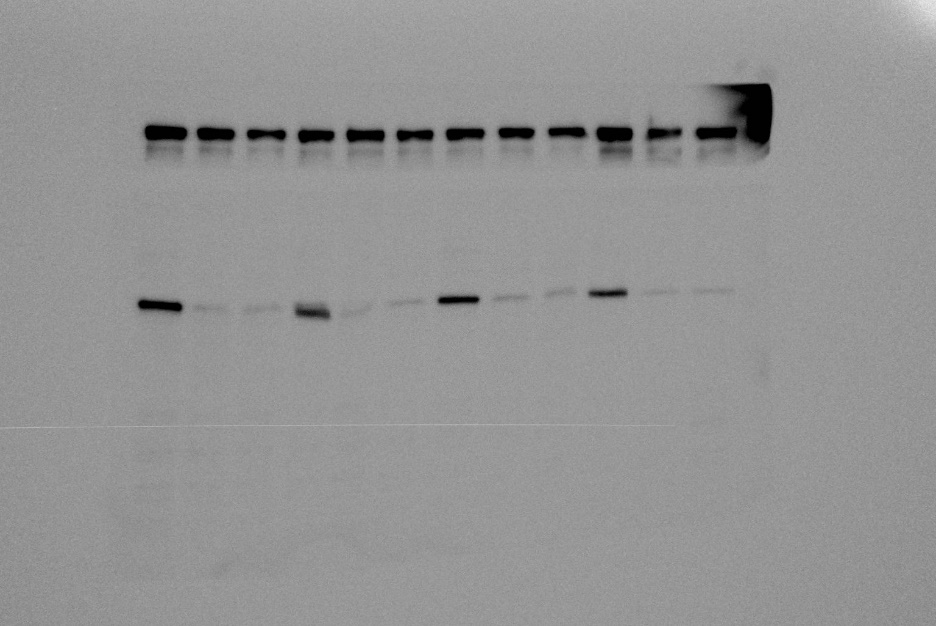


54 kDa

total-Akt
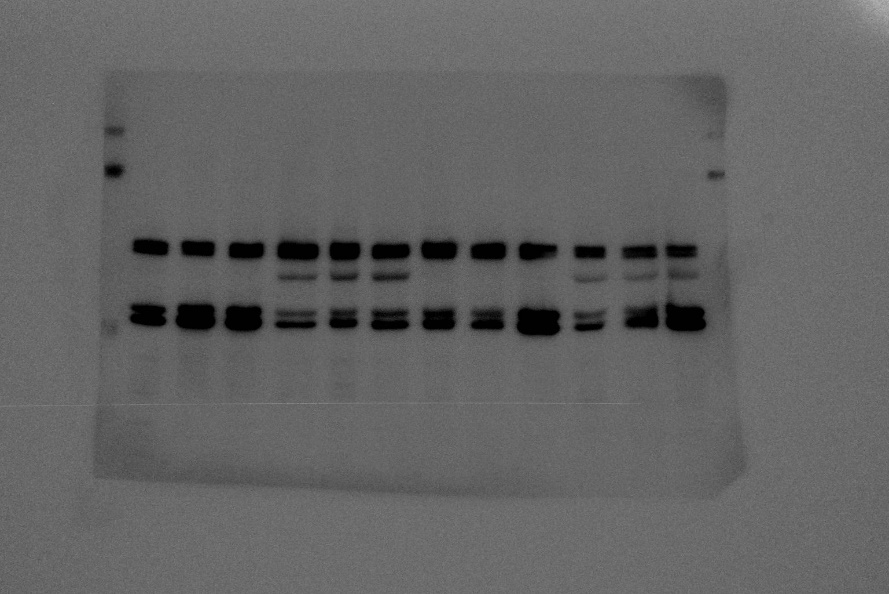


60 kDa

phospho-Akt
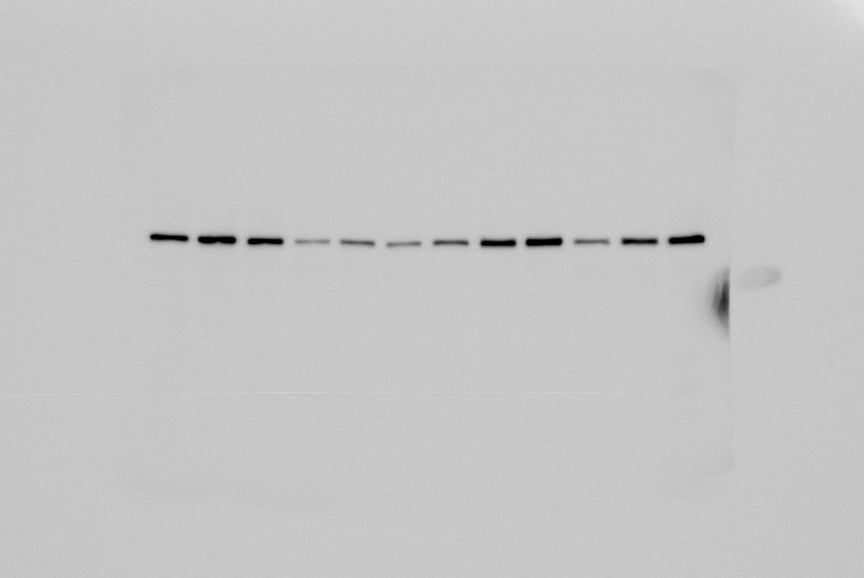


60 kDa

total-S6
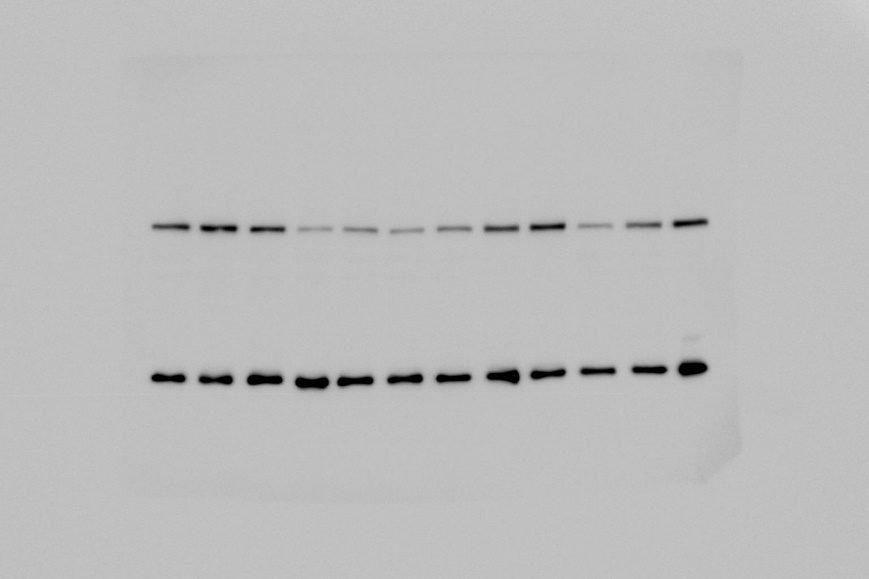


32 kDa

phospho-S6
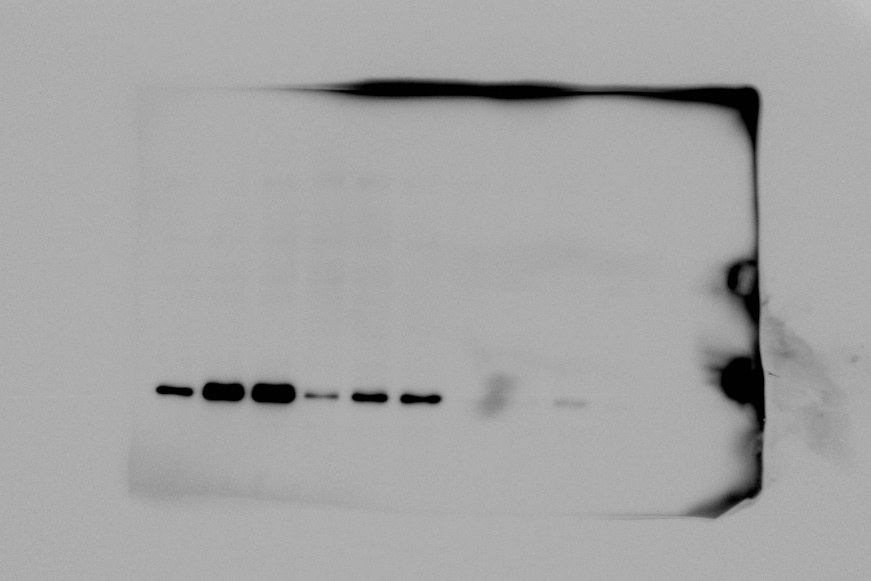


32 kDa

total-ERK
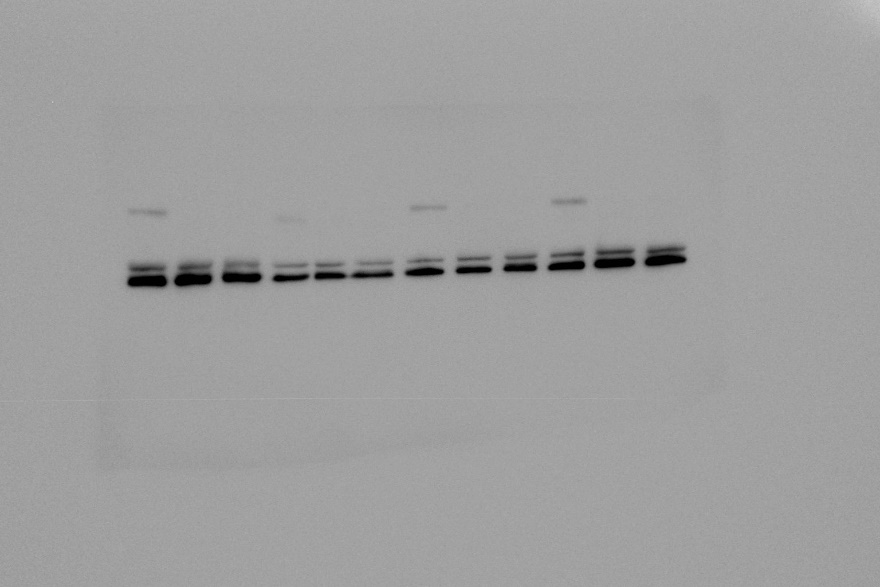


44, 42 kDa

phospho-ERK
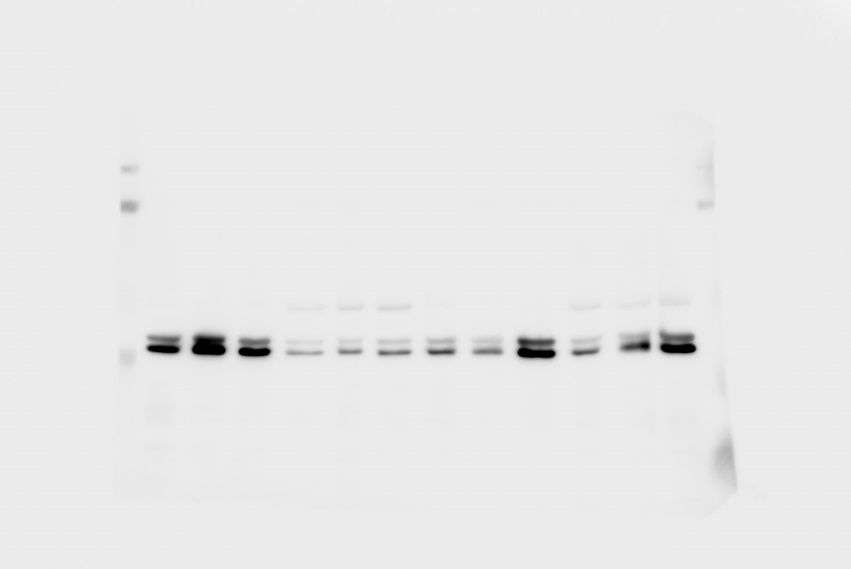


44, 42 kDa

ACTB
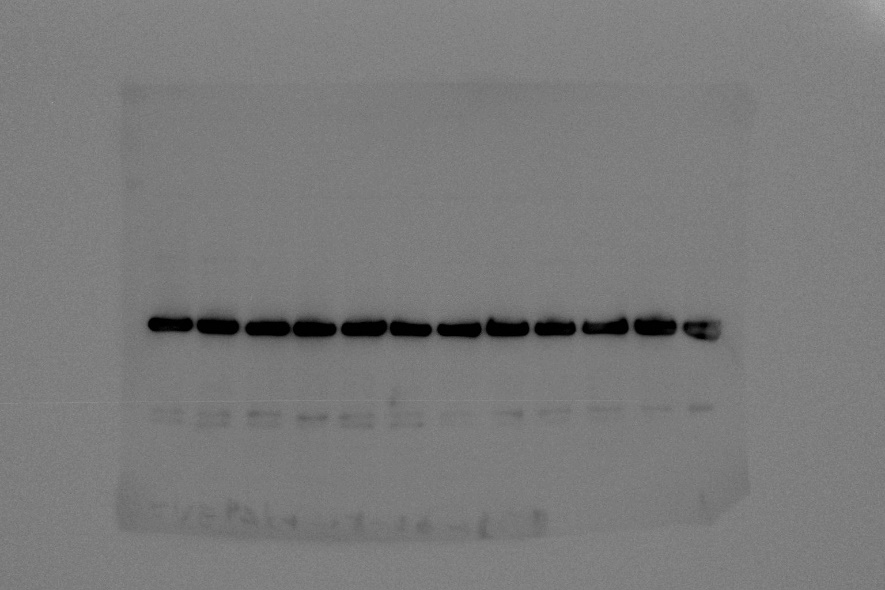


**MK2206**

HER2


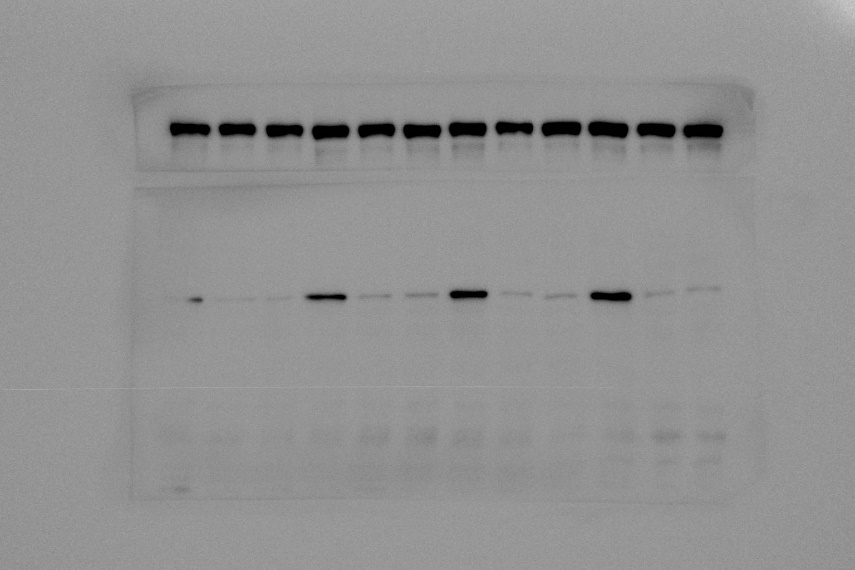


185 kDa

PTEN


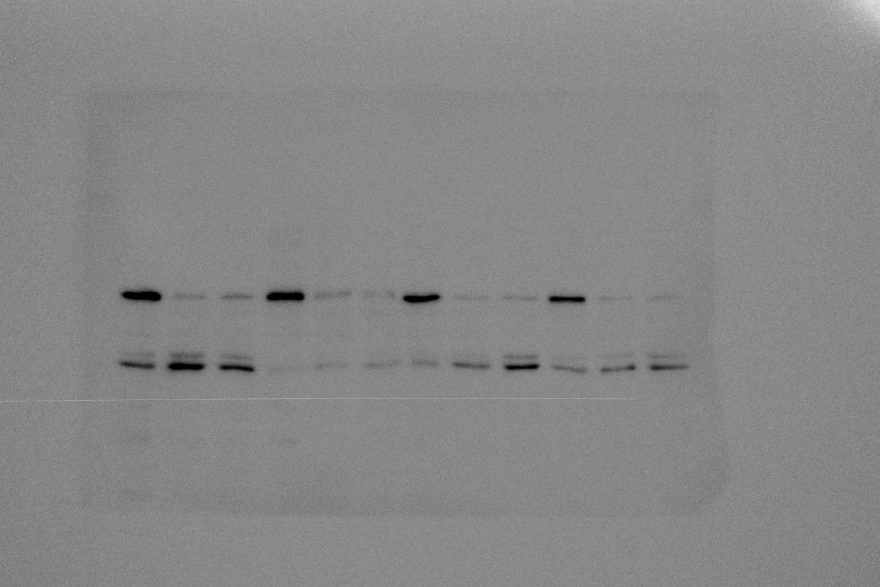


total-Akt
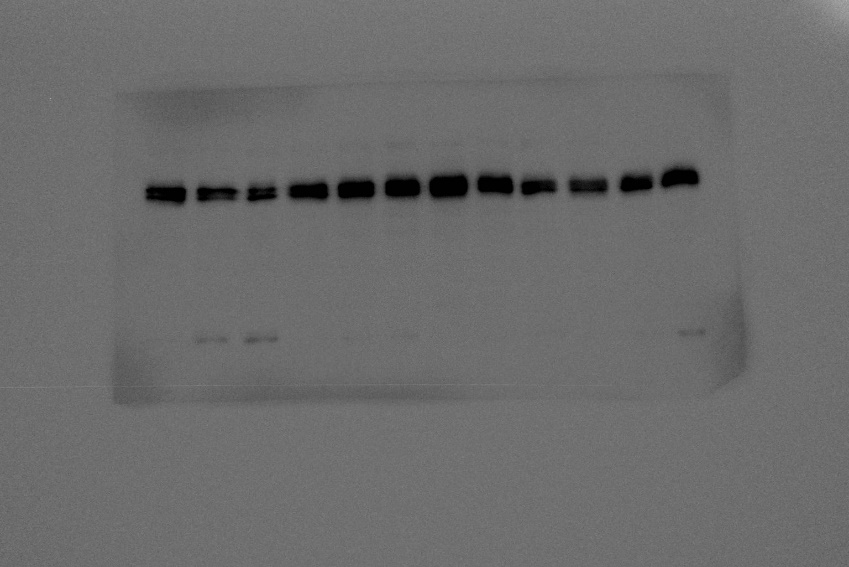


60 kDa

phospho-Akt
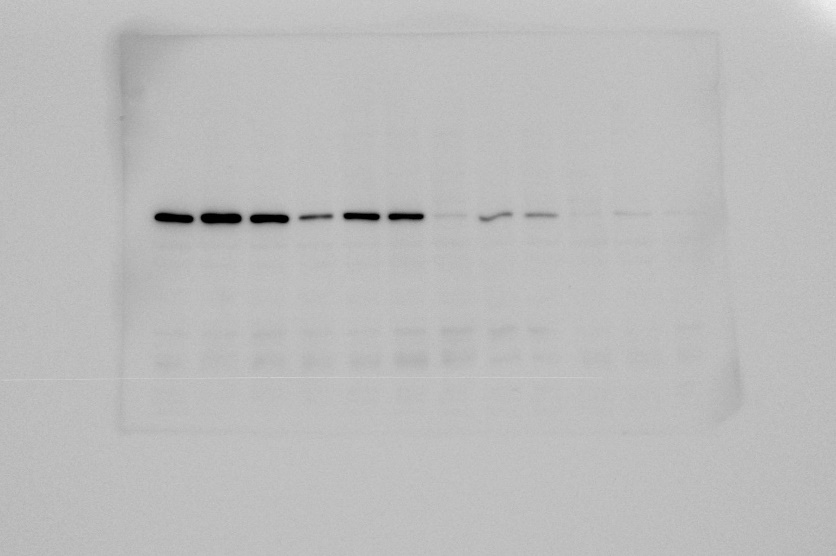


60 kDa

total-S6
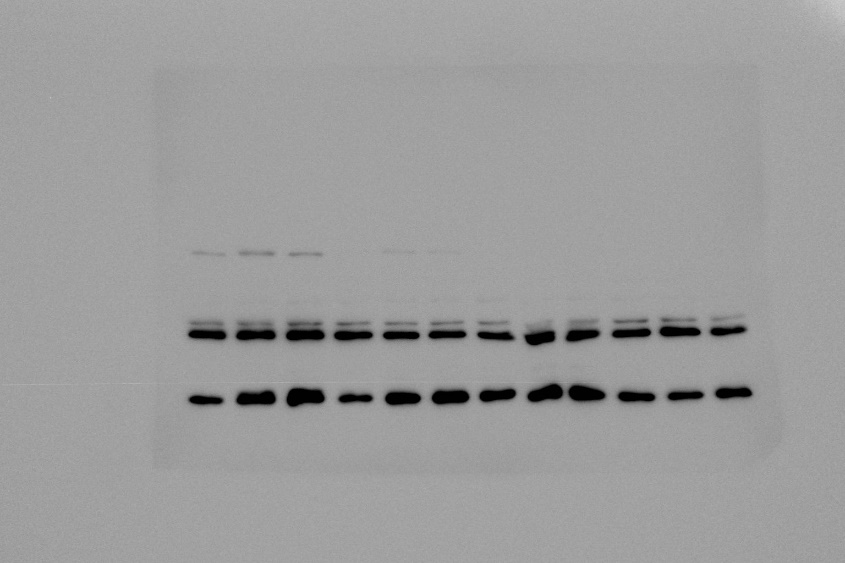


32 kDa

54 kDa

phospho-S6
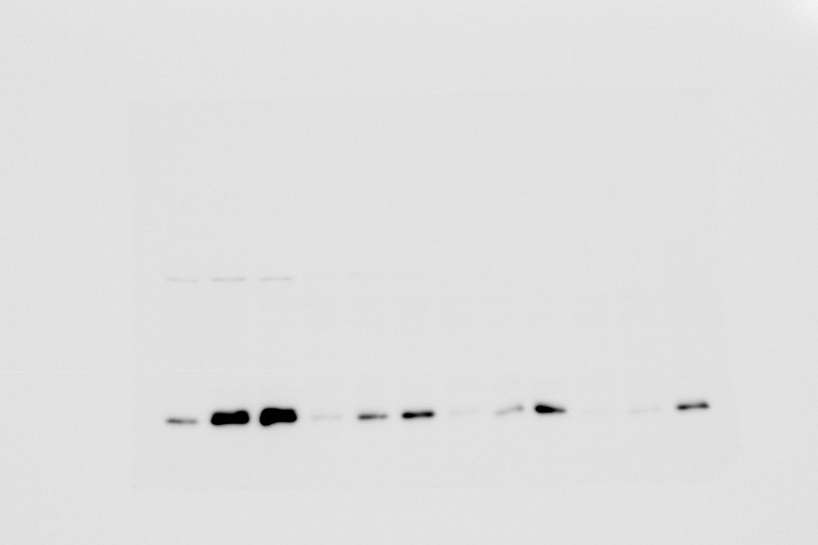


32 kDa

total-ERK
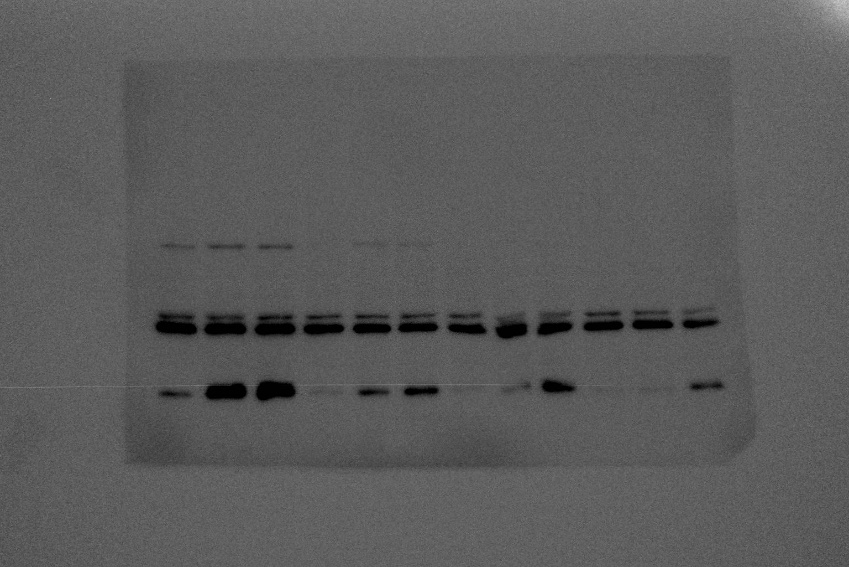


44, 42 kDa

phospho-ERK
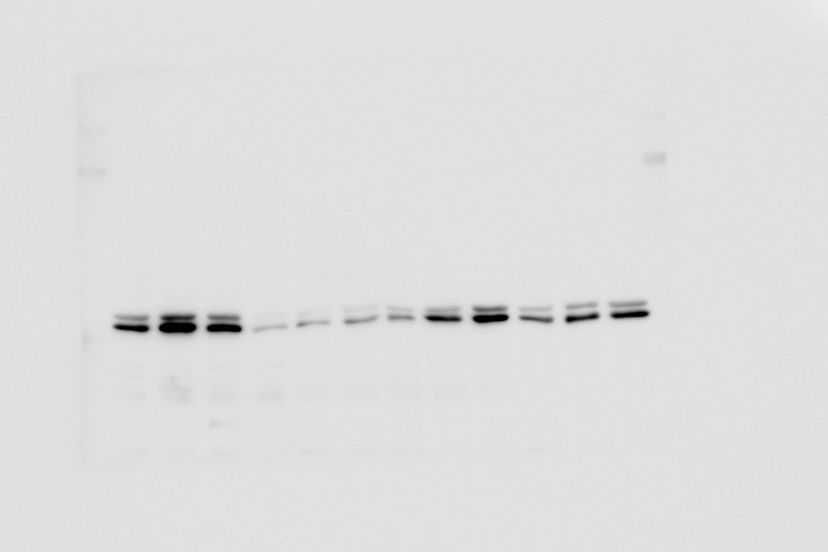


44, 42 kDa

ACTB


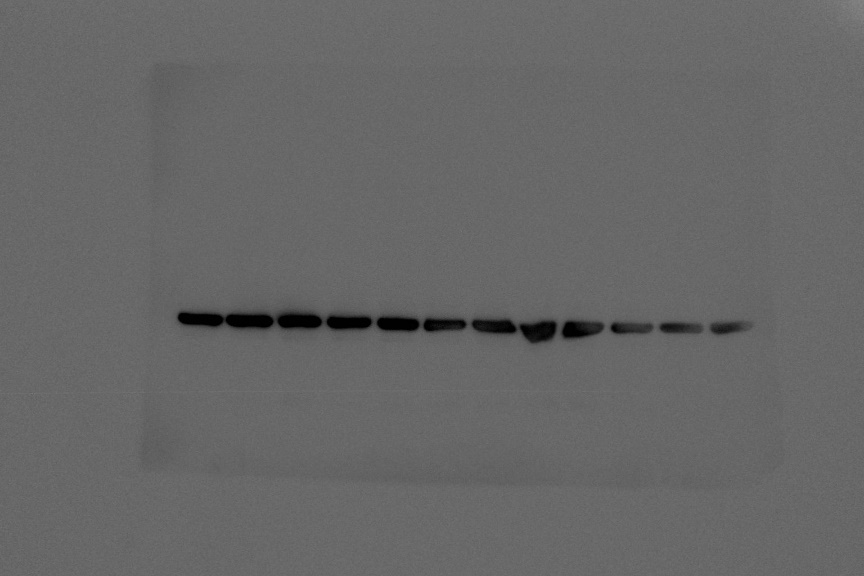


**NVP-BEZ235**

HER2


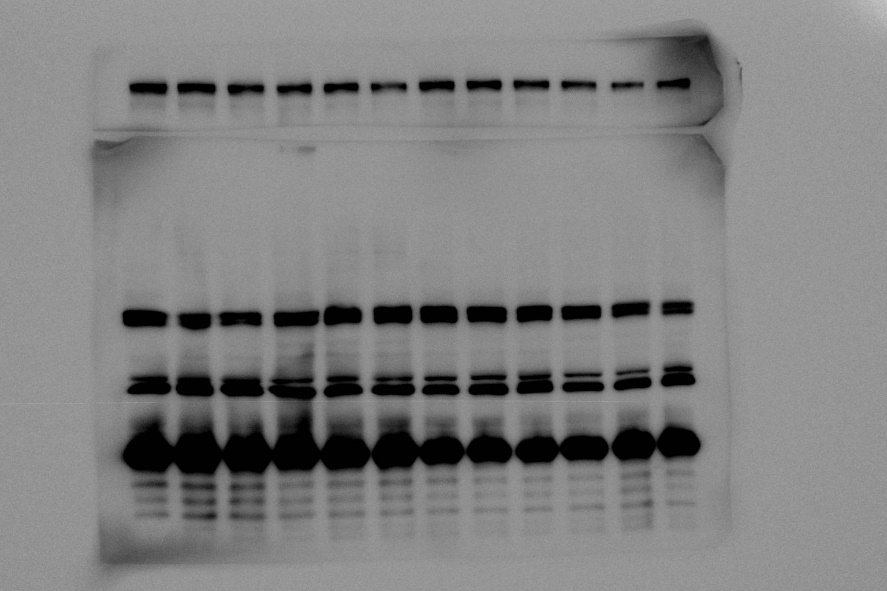


185 kDa

PTEN


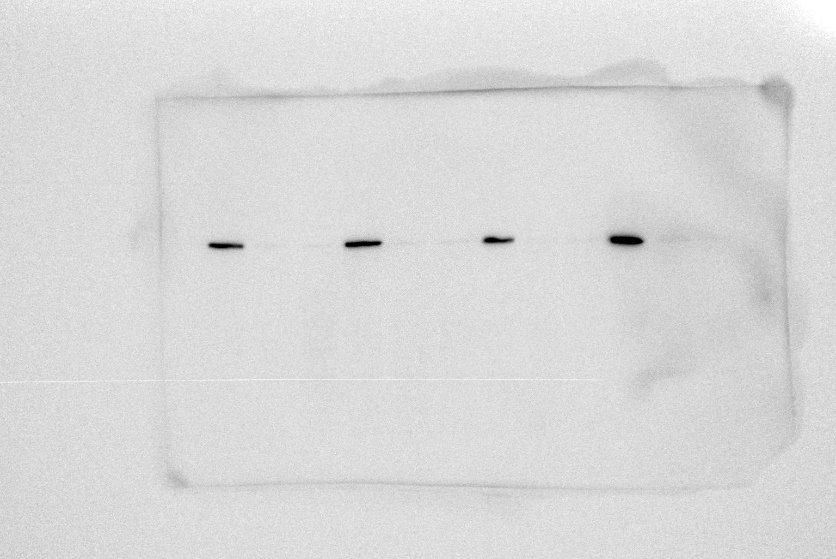


54 kDa

total-Akt
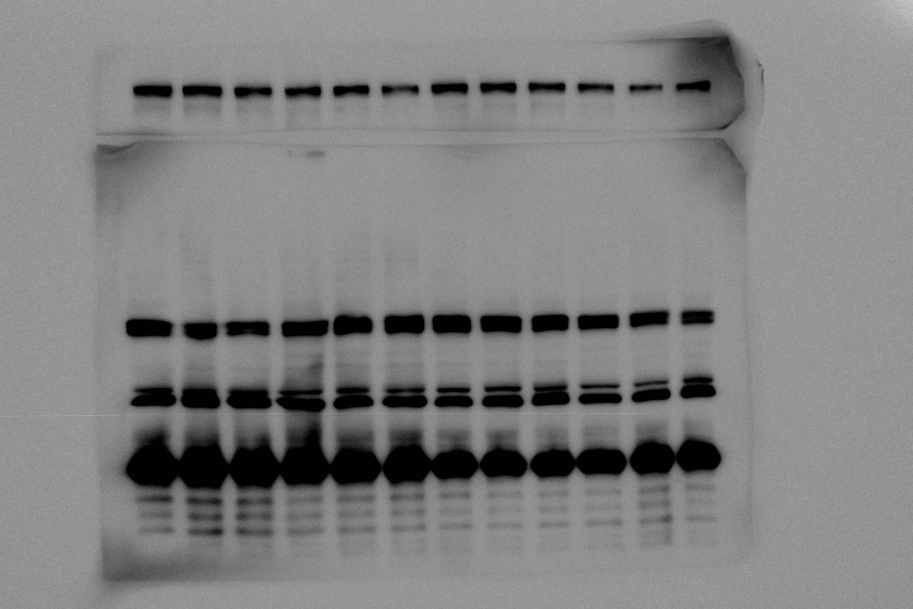


60 kDa

phospho-Akt
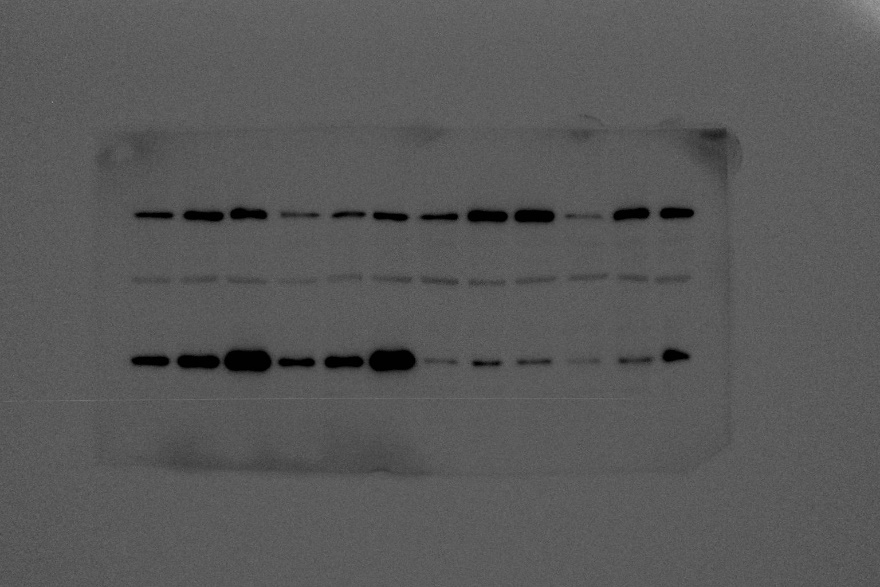


60 kDa

total-S6
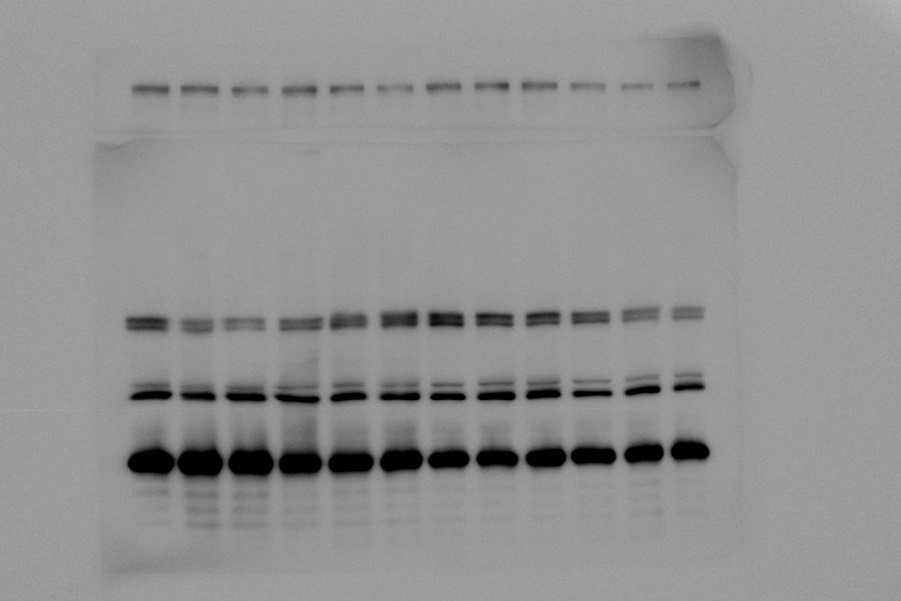


32 kDa

phospho-S6
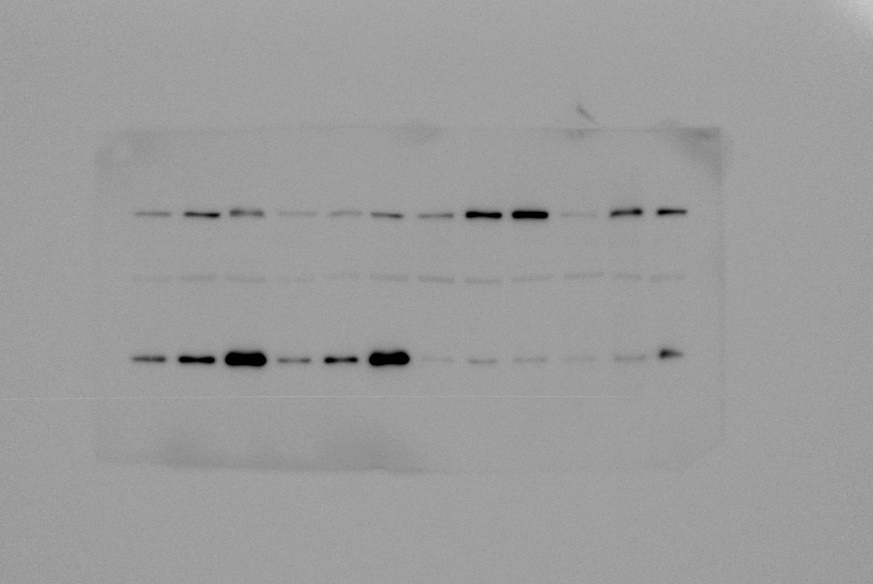


32 kDa

total-ERK
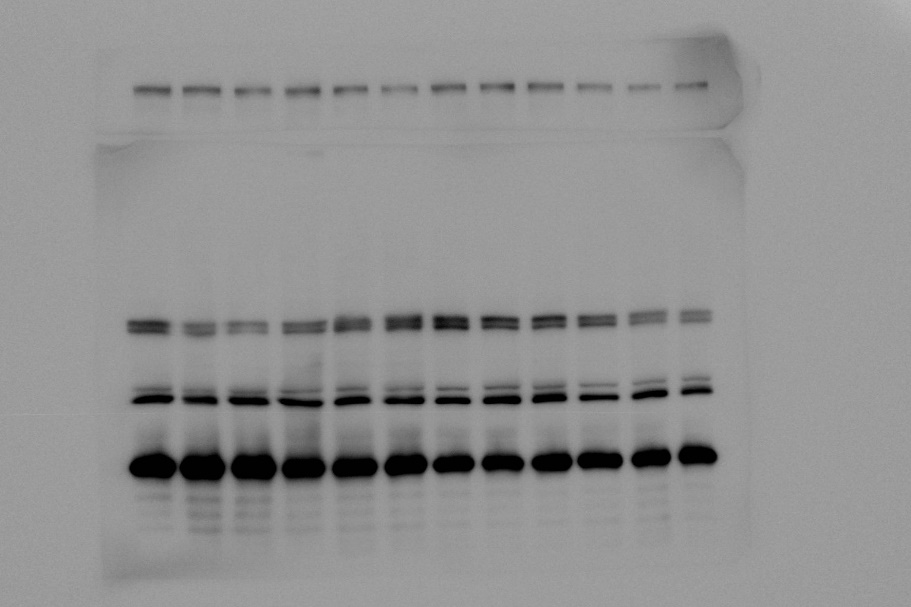


44, 42 kDa

phospho-ERK
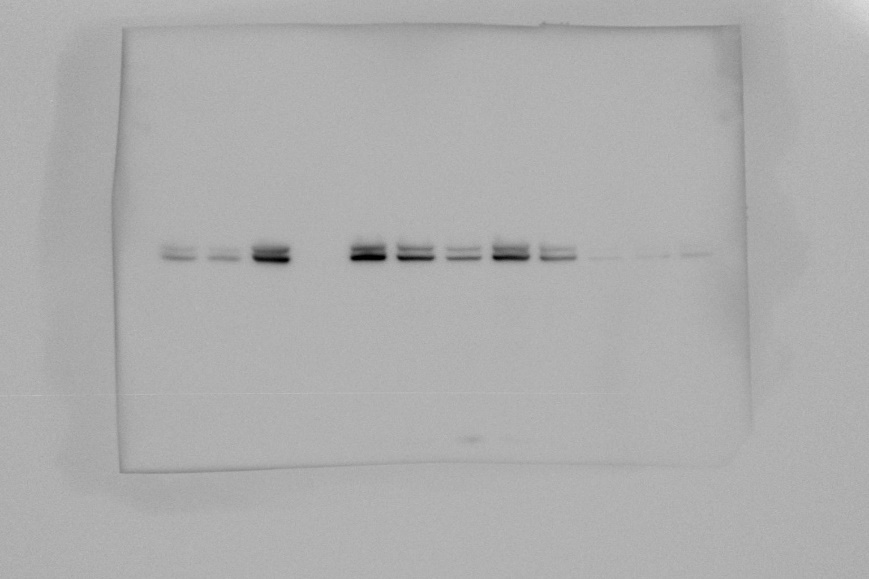


44, 42 kDa

ACTB


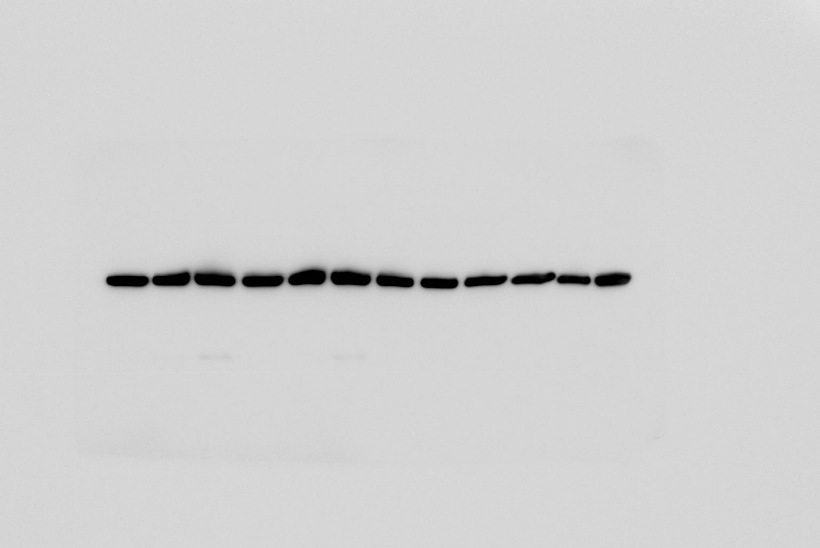


Original western blot images for Supplementary Figure 1

**N87**

HER2


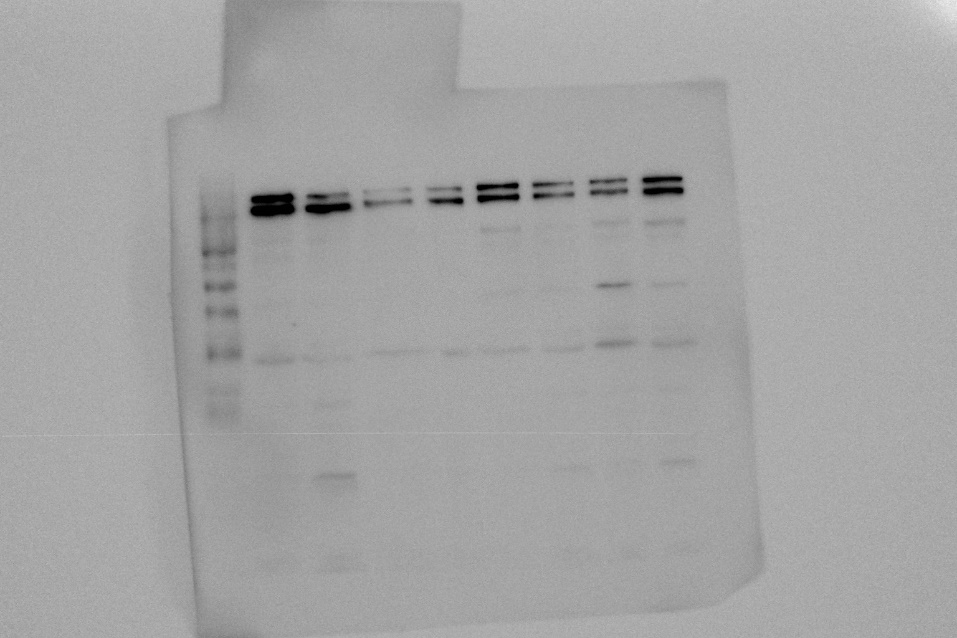


185 kDa

PTEN


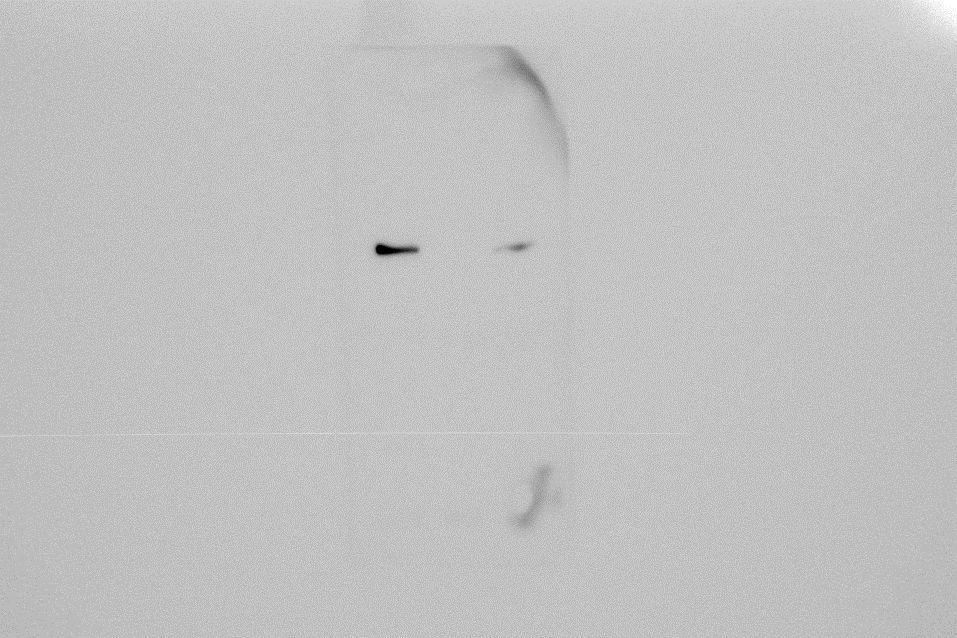


54 kDa

ACTB


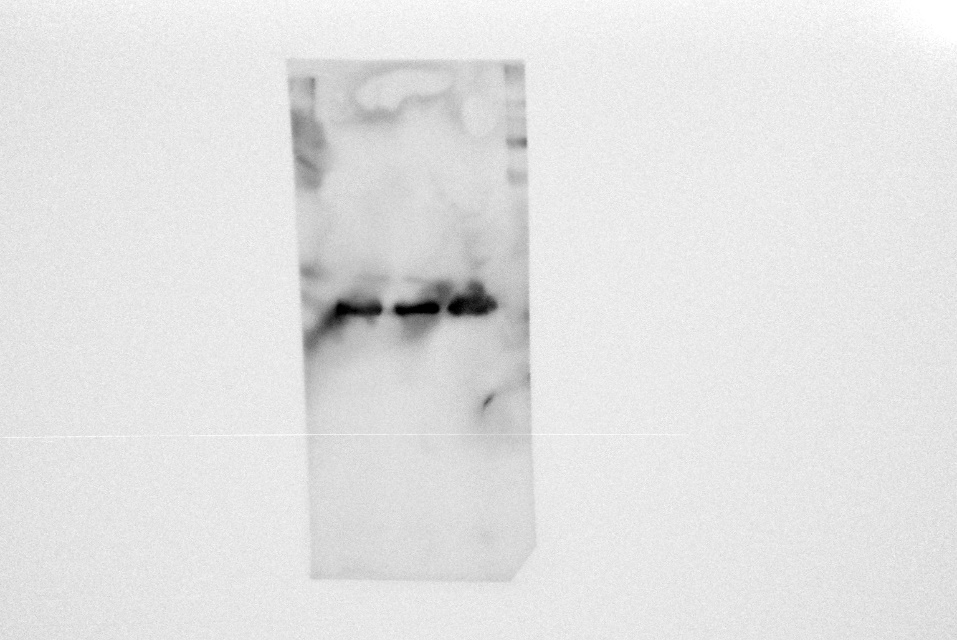


**OE19**

HER2


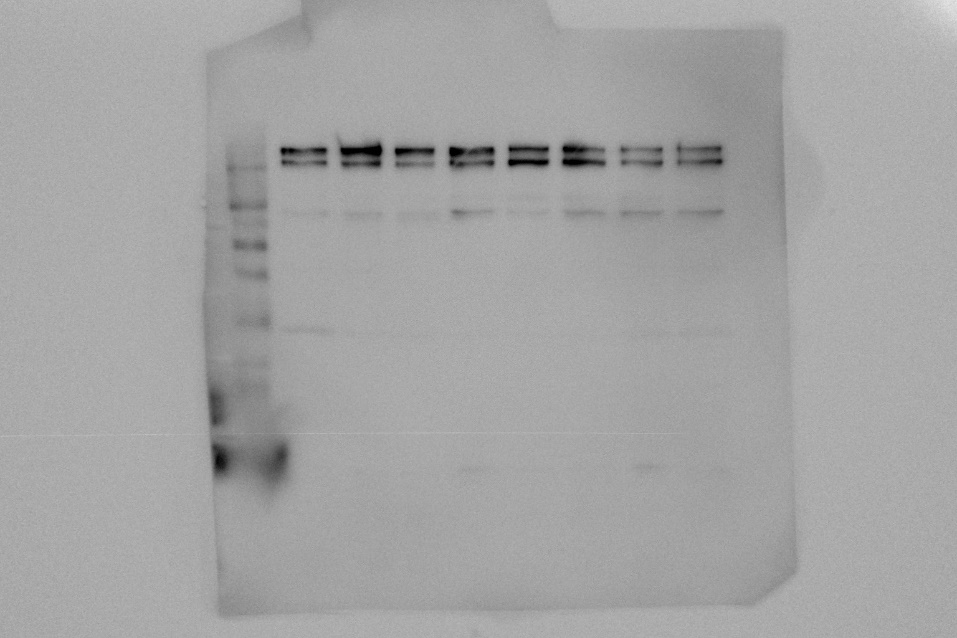


185 kDa

PTEN


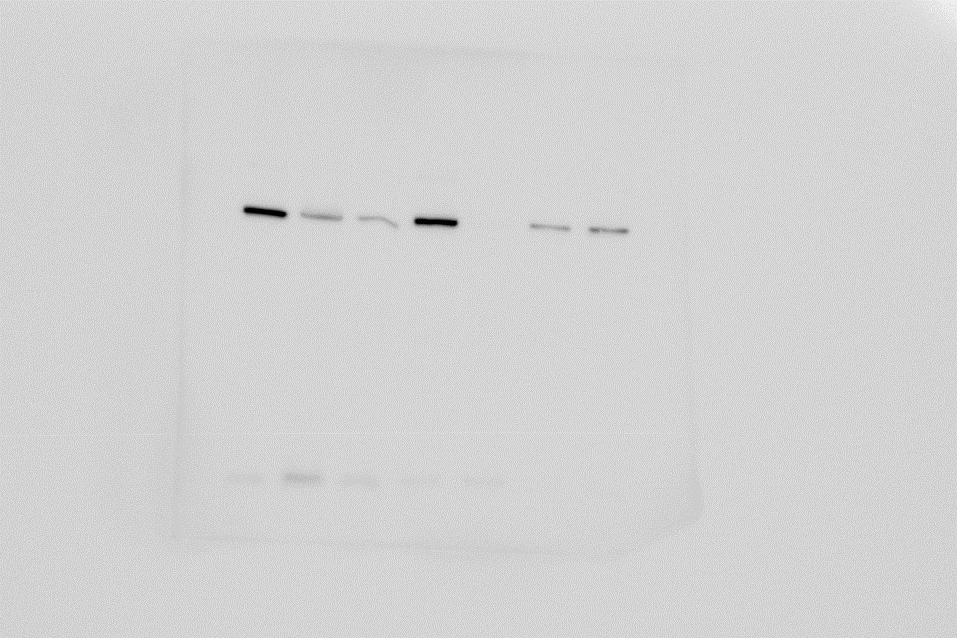


54 kDa

ACTB


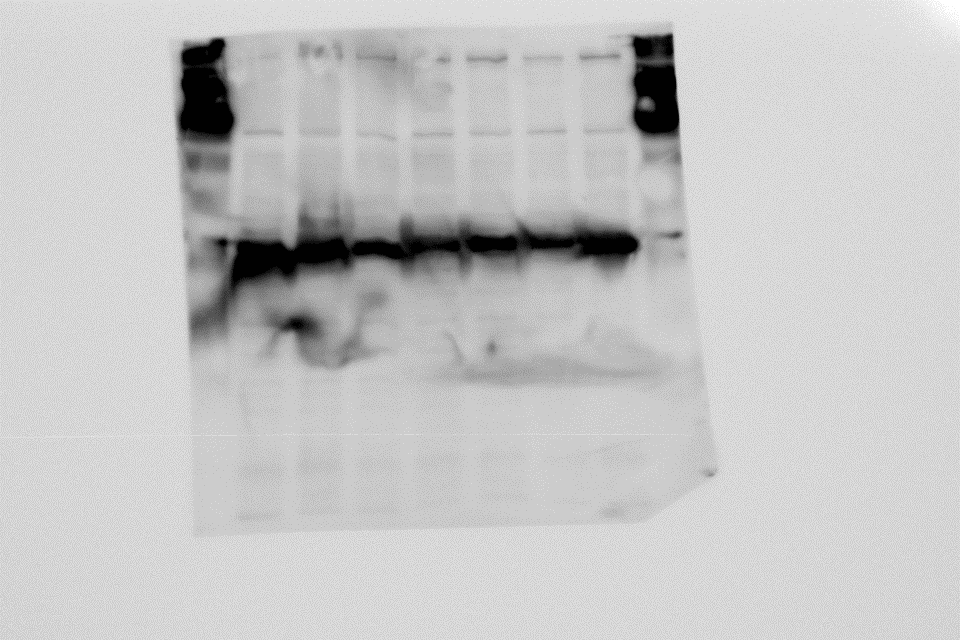

Supplement: Supplementary file 1 — Supplementary Figures. [file 41598_2021_88331_MOESM1_ESM.docx]
